# Hidden order across online extremist movements can be disrupted by nudging collective chemistry

**DOI:** 10.1038/s41598-021-89349-3

**Published:** 2021-05-19

**Authors:** N. Velásquez, P. Manrique, R. Sear, R. Leahy, N. Johnson Restrepo, L. Illari, Y. Lupu, N. F. Johnson

**Affiliations:** 1 Institute for Data, Democracy and Politics, George Washington University, Washington, DC, 20052 USA; 2 Theoretical Biology and Biophysics Group, Los Alamos National Laboratory, 87545 Los Alamos, NM Mexico; 3 Department of Computer Science, George Washington University, Washington, DC, 20052 USA; 4 ClustrX LLC, Washington, DC, USA; 5 Physics Department, George Washington University, Washington, DC, 20052 USA; 6 Department of Political Science, George Washington University, Washington, DC, 20052 USA

**Keywords:** Statistical physics, thermodynamics and nonlinear dynamics, Health care, Physics

## Abstract

Disrupting the emergence and evolution of potentially violent online extremist movements is a crucial challenge. Extremism research has analyzed such movements in detail, focusing on individual- and movement-level characteristics. But are there system-level commonalities in the ways these movements emerge and grow? Here we compare the growth of the Boogaloos, a new and increasingly prominent U.S. extremist movement, to the growth of online support for ISIS, a militant, terrorist organization based in the Middle East that follows a radical version of Islam. We show that the early dynamics of these two online movements follow the same mathematical order despite their stark ideological, geographical, and cultural differences. The evolution of both movements, across scales, follows a single shockwave equation that accounts for heterogeneity in online interactions. These scientific properties suggest specific policies to address online extremism and radicalization. We show how actions by social media platforms could disrupt the onset and ‘flatten the curve’ of such online extremism by nudging its collective chemistry. Our results provide a system-level understanding of the emergence of extremist movements that yields fresh insight into their evolution and possible interventions to limit their growth.

## Introduction

Online extremism often develops into offline violence^1–5^. The crowd that stormed the U.S. Capitol Building in January 2021 included members of extremist groups that use social media to coordinate activities, including members of the Boogaloo movement studied here^6^. A month ago, in February 2021, Canada became the first country to add the Proud Boys, another extremist movement with a prominent online presence, to its official list of terrorist entities^7^. A few weeks later, the FBI called attention to the rising threat of domestic terrorism in the U.S.^8^. Youngblood recently provided an analysis of 416 far-right extremists exposed in the United States between 2005 and 2017, discussing how social media usage and group membership enhance the spread of extremist ideology and concluding that online and physical organizing remain primary recruitment tools^9^. Online extremism and its recruitment activities pose a significant threat that could lead to real world terror threats^10–21^ and hence needs to be understood and mitigated^10–29^—regardless of whether the underlying movements are far-right, far-left, or occupy some other place in the political space.

Social media platforms are struggling to contain the growth of online extremist movements. Platforms often adopt a combination of content moderation and actively providing (or promoting users who provide) counter-messaging^23^. Much of the academic work in this area focuses on how to make these tools more effective^25–27^. However, while content moderation can be effective, it raises important concerns about censorship, and social media platforms are wary of being accused of political favoritism^21^. Moreover, counter-messaging is resource-intensive and, in some cases, counter-productive^24^. New strategies are therefore needed to complement these existing tools.

**Figure 1:**
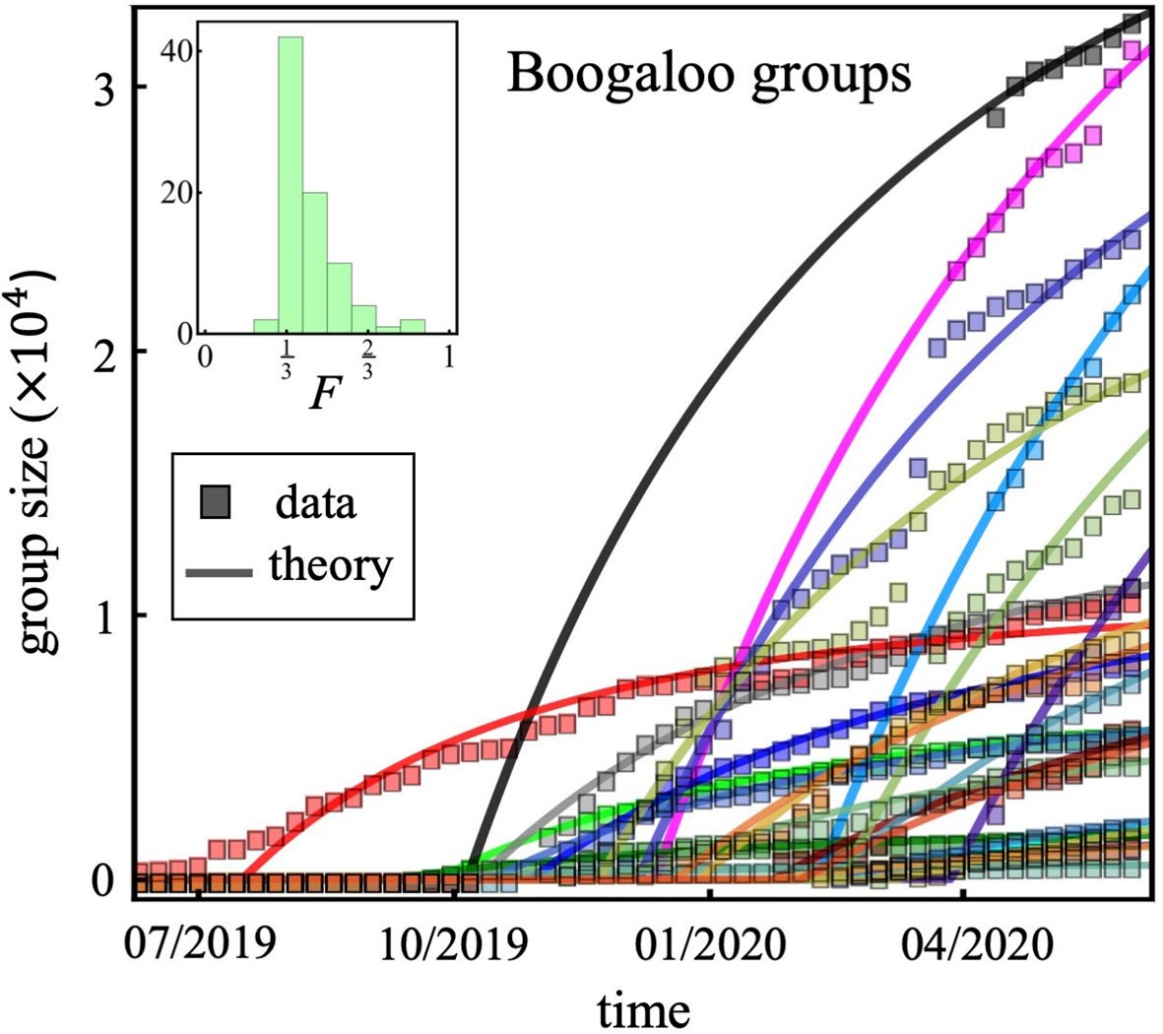
Growth curves of online Boogaloo groups. Each color/curve represents a Facebook Page that is a Boogaloo group, the size of which is the number of online members. Their empirical growths (symbols) differ and occur over different timescales. The solid lines show different allowed solutions of the same mathematical equation that incorporates individual user heterogeneity into the online social aggregation process (see Supplementary Information (SI) Eq. (21), with full derivation shown in SI Sect. 2). For visual clarity, only a few of the groups are shown in the main plots.

**Figure 2:**
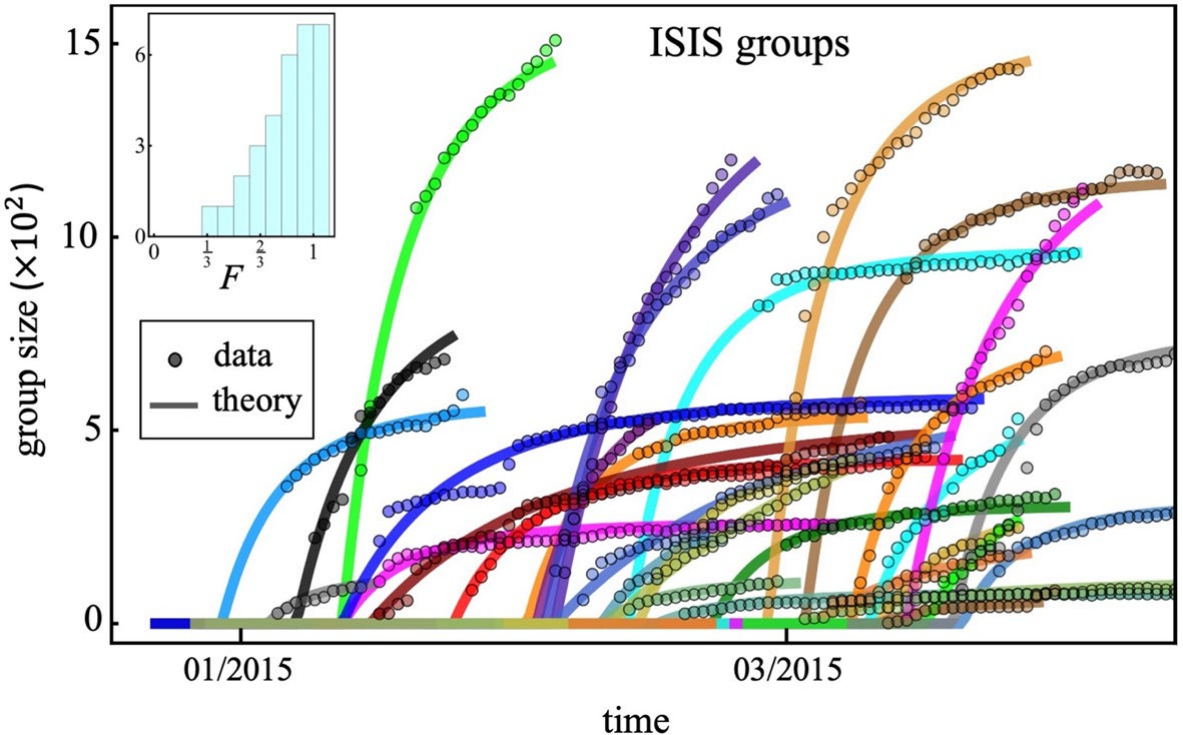
Growth curves of online ISIS groups, analogous to Fig. 1. Each color/curve represents a VKontakte Group that is an ISIS group, the size of which is the number of online members. Their empirical growths (symbols) differ and occur over different timescales. The solid lines show different allowed solutions of the same mathematical equation that incorporates individual user heterogeneity into the online social aggregation process (see Supplementary Information (SI) Eq. (21), with full derivation shown in SI Sect. 2). For visual clarity, only a few of the groups are shown in the main plots.

**Figure 3:**
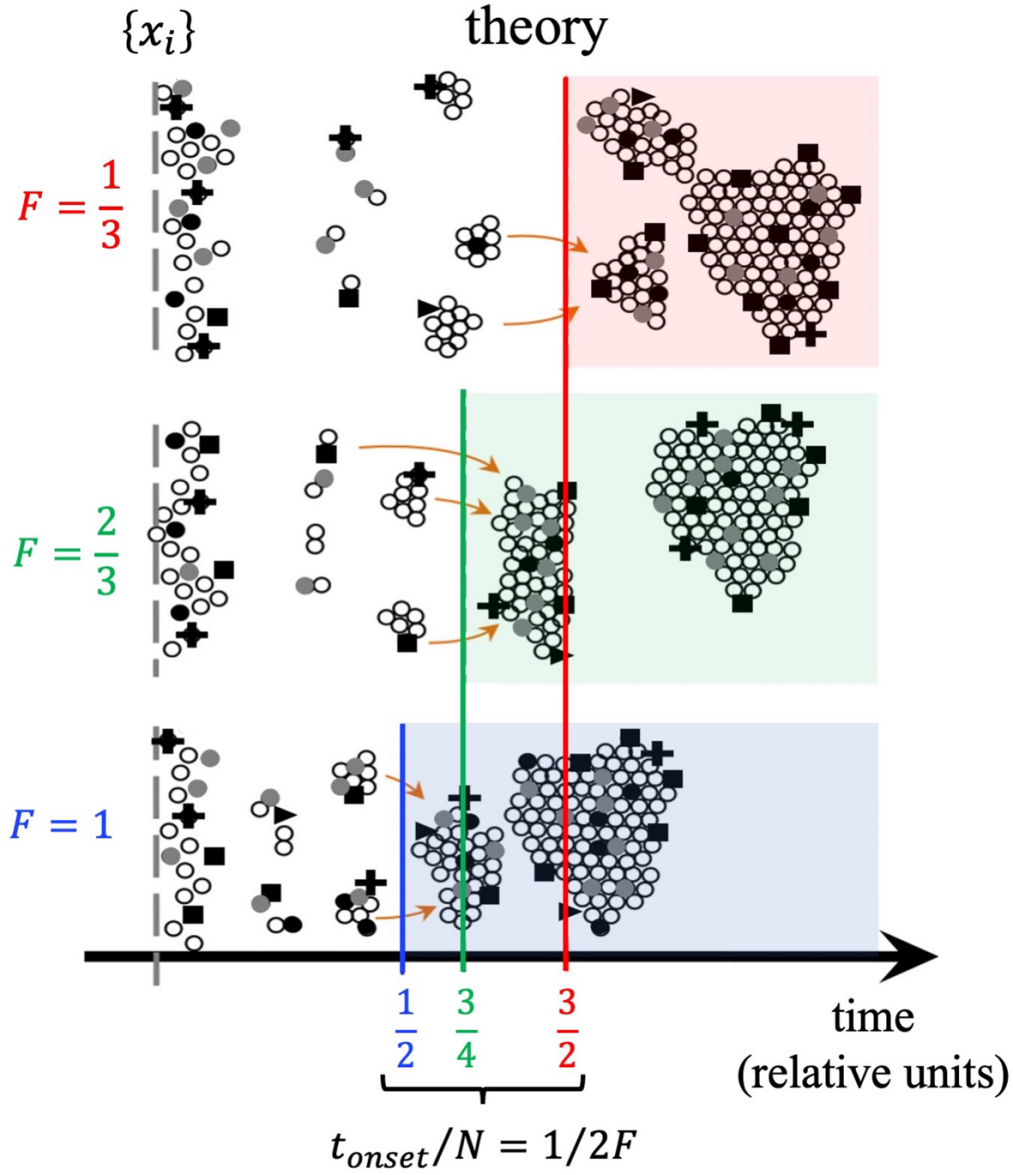
Collective chemistry. Sudden appearance of a large clump of correlated objects, which is referred to in the physical and chemical literature as a gel, or in the network science field as the giant connected component (GCC). This is shown for different values of the average aggregation probability *F*. *F* depends on the initial composition of the population (i.e. distribution of ${\overrightarrow{x}}_{i}$ values) and the grouping mechanism (see SI Sect. 2.2, Eqs. (1), (2)) and hence embodies the collective chemistry. For a uniform initial composition, an $F$ value of 1 indicates any individual could fit into any group, 2/3 indicates grouping via homophily, and 1/3 indicates grouping via heterophily. The horizontal axis time values are scaled so that ${t}_{onset}=N/2F$ appears as ${t}_{onset}/N=1/2F$, e.g. onset time for $F=2/3$ appears as $1/2F=3/4$. The accompanying mathematical theory is described in full detail in Sect. 2 of the SI.

**Figure 4:**
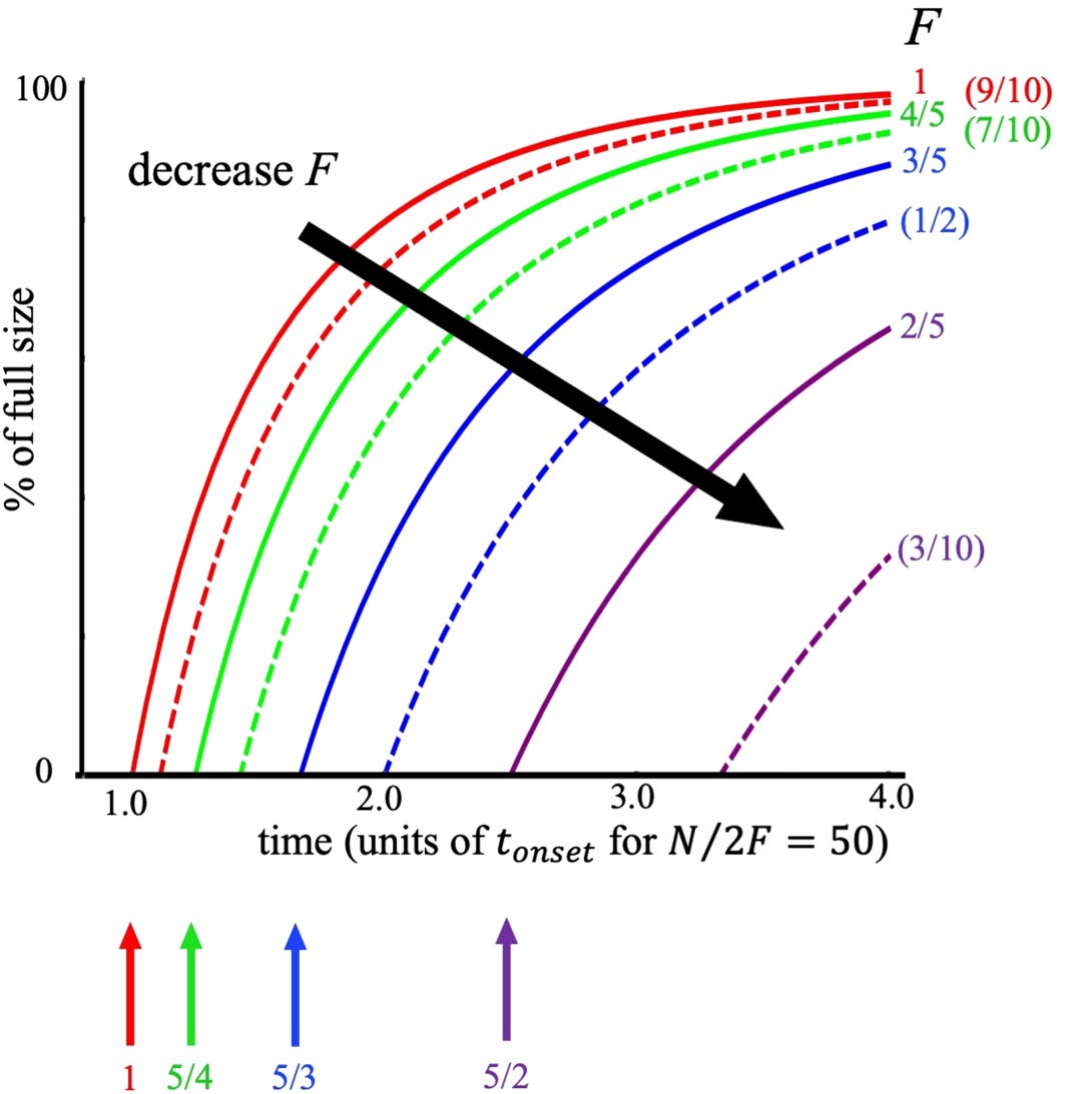
Predicted curves from our theory showing the onset and growth of a gel (i.e. each online group in Figs. 1 and 2, or each overall movement in Fig. 5) and how it depends on the average aggregation probability $F$. The specific $F$ value is determined by the initial composition of the population and the grouping mechanism, i.e. the collective chemistry, hence we show a variety of possible values as examples. An intervention to this collective chemistry will change $F$ and hence can delay the onset of the gel as shown and curtail its growth. Specifically, the curve can be flattened by reducing the value of $F$, i.e. by nudging the collective chemistry.

**Figure 5:**
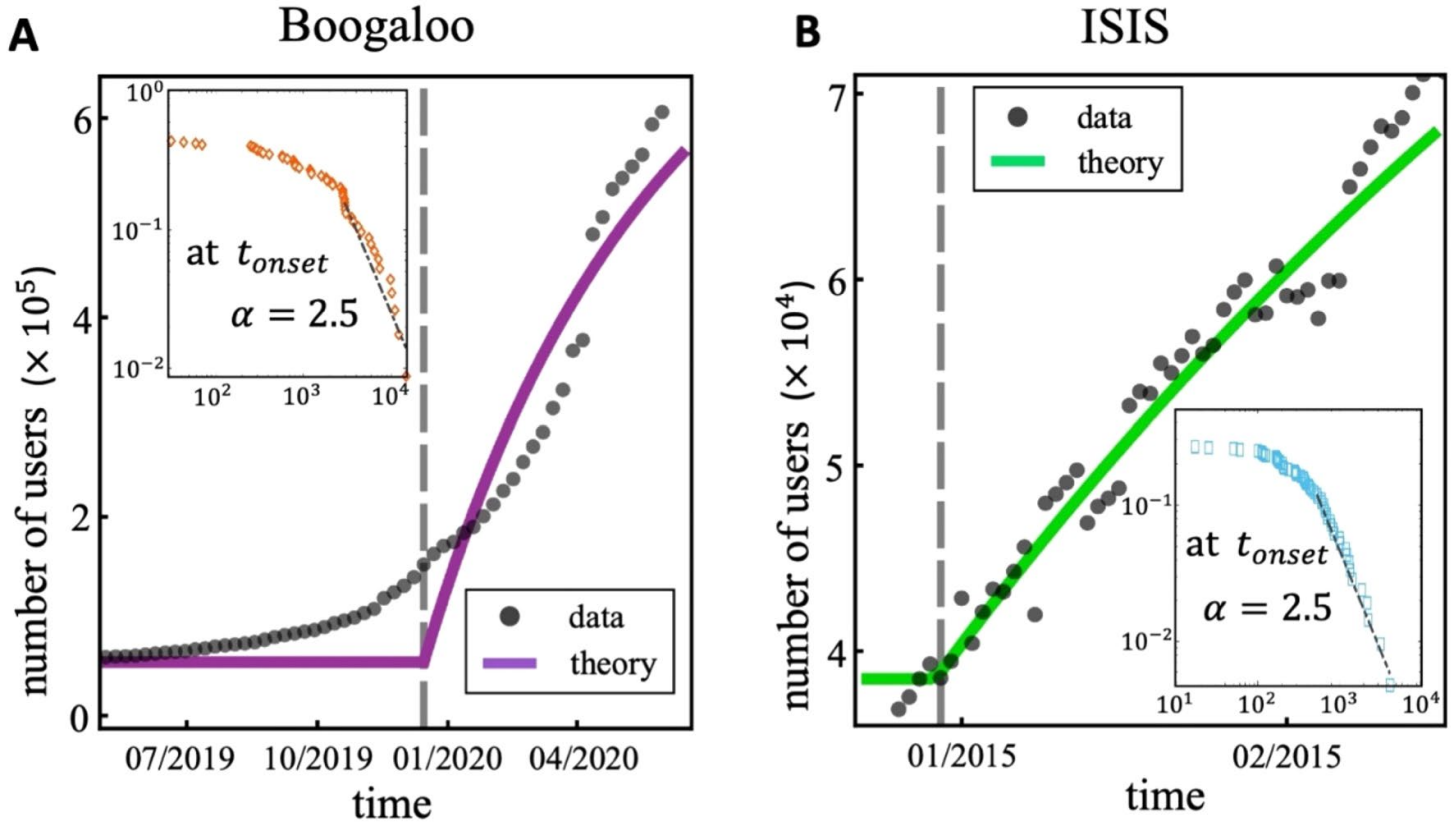
(**A,B**) Growth curves of the overall Boogaloo movement (size is the combined number of users in their Facebook Pages), and the entire ISIS movement (size is the combined number of users in their VKontakte Groups). Insets show distribution of sizes of the individual groups at the predicted onset ${t}_{onset}$ (vertical gray line in main panels). The maximum-likelihood estimate for the magnitude of the negative power-law exponent in each case is $2.5$ to two significant figures, which is the same value as predicted by our mathematical theory (SI Sect. 2.7, Eq. (23)). The rigorous statistical analysis that we follow to obtain these results, is given in SI Sect. 3.1.

This paper provides a quantitative study of the emergence of such movements online: in particular, we study how the groups of online supporters emerge and grow over time (Figs. 1, 2, 3, 4, 5). We purposely take a physical science approach in order to build a mathematical description, but with an important generalization that accounts—albeit in a necessarily simplistic way—for the heterogeneity of the human population that joins such online movements. This work therefore builds on the physics, chemistry, and mathematics literature, with the generalization that particles (individuals) that are typically treated as identical can now be different, and this can affect how they form groups (e.g. via homophily). This approach makes the mathematical aspect of our research potentially of interest in its own right, in addition to the proposed application.

We refer to the model framework as “collective chemistry” because the group dynamics depend on the attributes of the population collectively, and in particular the relationships between the attributes of existing members and the attributes of potential new recruits. This is illustrated in Fig. 3. The onset of the group's growth and its growth shape depend on the average aggregation probability *F* as illustrated in Figs. 3 and 4, and *F* in turn encapsulates this collective chemistry. Prior literature has accommodated heterogeneity in models of human behavior in other settings using either a vector or scalar quantity to account for individuals' traits and characteristics^30,31^. Centola et al.^30^ discuss in depth how such a simple mathematical approximation is nonetheless consistent with a line of successful earlier works in sociology by Axelrod and others. Drawing on the physics and chemistry literature, we refer to the groups that form as gels, or equivalently as the giant connected component (GCC) in a network setting.

We compare two online movements that differ markedly in terms of their ideology, institutional structure, geographic base, and aims. The U.S.-based Boogaloos are a loosely organized, pro-gun-rights movement focused on preparing for, and some instances inciting, what its members believe is a coming civil war in the U.S., with members drawn from diverse conservative, libertarian, and nihilistic ideologies^1–3^. This movement emerged online and first came to public prominence in 2020, and its members have already been implicated in recent violent crimes in the U.S.^1–3^. ISIS, by contrast, adheres to a specific ideology, a radicalized form of fundamentalist Islam, and has a formal leadership and hierarchy. It initially organized offline and later used social media to gain followers, is based in the Middle East, seeks to establish itself as a formal state with authority over all Muslims, and has claimed responsibility for terrorist attacks across the world.

We stress that our goal is not to provide a philosophical, psychological, economic, or sociopolitical analysis of such movements, but rather to elucidate the possible mechanics of their online growth by comparing a mathematical model of aggregation to empirical data (Figs. 1, 2, 3, 4, 5). Clearly the movements we study are very different in their ideology, origins, and goals, so a comprehensive description of them requires both a quantitative and a qualitative discussion. The sociological reasons for why and how social movements grow, offline and online, can likely be explained in part by individual-level factors, such as poverty and personality traits, as well as movement-level factors such as ideology, culture, and political aims^10–20^. Yet system-level factors are also crucial, and recent work suggests that these play important roles in the development and countering of online extremism^22,28,29^. We are motivated by the notion that, despite their differences, extremist movements may share common system-level dynamics. We therefore take a modest route of focusing on possible shared mechanics of their online growth processes, with the purpose of identifying any common mechanical patterns that might arise^32–37^ and seeking a better understanding of how online violent movements emerge and grow. Here we explore such system factors in a simple, quantitative way which, in turn, suggests new countering strategies to complement content moderation and counter-messaging.

## Methodology

### Data collection

We collected our data by manually observing public online communities: Facebook Pages for Boogaloos, VKontakte Groups for ISIS. We follow the methodology described below, which was first developed and used in Refs.^38–42^. Such communities are known to facilitate coordination and play a greater role in nurturing narratives than platforms like Twitter, which have no pre-built community tool and are instead designed for broadcasting short messages. For clarity, we refer in this paper to the Boogaloos and ISIS supporters each collectively as a “movement,” and to the online communities that support them as “groups.”

We collected the data on online pro-ISIS groups in 2014–2015, during the movement’s growth period. On a daily basis during that period, we manually searched for groups using common hashtags and keywords. This list of groups for each day was updated to include only those appearing to express a strong allegiance to ISIS. Since no such support was observed on Facebook, likely as a result of its being removed quickly, we looked on another social media platform based in Europe, VKontakte (www.vk.com), which was comparatively slow in removing ISIS support. The daily search for newly created pro-ISIS groups was achieved by (a) analyzing posts and reposts within the known pro-ISIS groups; and (b) following selected profiles that actively published ISIS news and analyzing the pro-ISIS groups that they followed, if any. Whenever a new group name was found, it was analyzed to establish the relevance of its narrative content; if found to be relevant, that group was included in the database.

Once the groups supporting ISIS were identified, an additional search for new links was performed on that same day. The manual content analysis helped identify newly created groups, as well as those that had been shut down. The following examples illustrate some of the types of content of the pro-ISIS groups we identified: (1) evidence of fundraising: Multiple incidents of collecting funds for potential fighters who wanted to travel to Syria but could not afford it. Also transfer of funds for fighters who were already in Syria. (2) Evidence of real-time operational information stream: Some groups resembled an alternative news outlet where they streamed information directly from their territory. Operational updates from battlefield, e.g. the specifics of a Kobane-based radio tower in real-time. One example image that we uncovered says “ISIS took control over the Kobane’s radio tower”; “Mujahedeen advanced 500 m into Kobane”. (3) Evidence of mobilizing support: Images include text such as: “Brothers! Yesterday, in a German town of Celle, a 100-people mob of Yazidi Kurds beat up 5 Chechens in retaliation for Chechens fighting within ISIS in Iraq and Syria where they kill Kurds. Since Celle has the largest Yezidi Kurds community (about 5000 people), our local brothers’ lives are under threat. This is a call to all brothers from nearby locations to send groups of 30–40 people to protect our brothers in distress”. (4) Teaching survival skills: Some pro-ISIS groups included advice on cellphone and Internet use during an operation in order to avoid being detected by security services, and also ways to prevent or repel a drone attack during an operation. (5) Evidence that the online groups serve as a platform to spread recruitment messages is illustrated by an example that states: “IS fighters in Dagestan call other Caucasus mujahedeen enter their ranks”. Indeed many Caucasus guerrilla groups joined ISIS later.

We collected the data on the Boogaloo groups in 2020 using a similar methodology, on Facebook because Boogaloo discussion was allowed on the platform at that time. We started by querying keywords associated with the movement, such as “boogaloo”, “b00g”, and “big igloo”, in Facebook’s search engine during the first week of May 2020. We limited the search results to publicly accessible Facebook Pages; specifically, we searched fan pages, rather than often-private Facebook Groups. We also avoided individual accounts in order not to violate Facebook’s Terms of Service. We checked the first 40 results, classifying as Boogaloo those Facebook Pages that (1) self-identified as such; and/or (2) identified as a local militia but which also used the Boogaloo movement’s iconic Hawaiian-inspired aesthetic paired with the establishment of local militias, or which claimed a connection with 4Chan’s /k/board and the Boogaloo movement.

### Our theory of online aggregation

In order to avoid interrupting the description of what the model means, we describe our mathematical model in words in the main paper and refer to specific formulae and sections in the SI for their derivations where necessary.

Our mathematical aggregation theory considers the emergence of online groups from a population of online users who we model as interacting, heterogeneous individuals. The details are laid out in Sect. 2 of the SI and are shown schematically in Fig. 3 as well as SI Figs. S1–S3. The SI Fig. S4 confirms the accuracy of our mathematical results, by comparing with stochastic computer simulations. Our mathematical analysis generalizes a long tradition of aggregation models in the physical sciences in which all particles are traditionally assumed identical^43–47^—hence we refer to it as a generalized aggregation theory. Specifically, it involves writing down a set of coupled rate equations for the number of these small clumps of individuals having size 1,2, etc. at any instant in time (SI Sect. 2, Eq. (3)). Including the effect of aggregation of these clumps, leads to the prediction of a transition to a phase with a gel at time ${t}_{onset}=\frac{N}{2F}$ where $N$ is the online pool size of potential recruits (SI Sect. 2, Eq. (15)). The formation of such a gel means that a significant fraction of all the individuals are in the same, large clump—which is termed a gel in the physical and chemical literature.

This generalized model can equivalently be viewed as applying to the linking together of objects in a network, or in a more abstract way as pockets of coupled or correlated entities. The gel—or, equivalently, the giant connected component (GCC) of the network—then emerges as a result of aggregation (SI Sect. 2, Eq. (20)). We apply this aggregation theory at 2 different scales in the main paper: (1) the emergence of a single Facebook Page (for Boogaloos) or VKontakte Group (for ISIS) is described as a gel that forms from within the movement itself as in Figs. 1 and 2 of the main paper; and (2) the emergence of each overall movement is described as a gel forming from the background pool of users on the Internet as in Fig. 5. The precise procedure that we employ for comparing the theory to the data is given in SI Sect. 3.2.

The novel feature of this mathematical theory of group formation is that it incorporates individual human heterogeneity into the online aggregation process (SI, Sect. 2.2). It mimics the heterogeneity of each individual $i$ by a vector ${\overrightarrow{x}}_{i}$, which can be of arbitrary dimensionality (i.e., any number of individual attributes, such as personality traits) and can in principle change over time. The dimensionality is the number of personality traits. The values of the elements in the character vector ${\overrightarrow{x}}_{i}$ could in principle by any value, but since they mimic a trait we take them as between 0 and 1.

The interaction between individuals is described in terms of their similarity or dissimilarity (diversity) and hence is a function of their respective ${\overrightarrow{x}}_{i}$ values (SI, Sect. 2.2). For simplicity, consider the one-dimensional case. We define the similarity ${S}_{ij}$ between individual $i$ and individual $j$ as ${S}_{ij}=1-\left|{x}_{i}-{x}_{j}\right|$, so that individuals with like character have a high similarity, and otherwise for a pair of individuals with unlike character. We consider that the probability of aggregation for any two individuals $i$ and $j$ under homophily as a group formation process, is given by ${S}_{ij}$. Our definition also recognizes the opposite mechanism of heterophily (diversity, dissimilarity) which tends to form clumps of dissimilar individuals, where the aggregation probability depends on ${1-S}_{ij}$. The random case is recovered in the limit where the aggregation probability is independent of individual character and hence always 1. Doing this in different dimensions leads to a set of diverse types of gel emerging, as observed empirically. The heterogeneous aspect of the aggregation process is then transferred to the equations for the evolving population by means of a population-level (so-called mean-field) average for this aggregation probability, which we call $F$ (SI, Sect. 2.3, Eqs. (3), (4)). Different values for $F$ will follow according to the choices made for the initial composition of the population and for the grouping mechanism (e.g., homophily, heterophily). Here we assume the simplest, most parsimonious choice for the initial composition: a uniform distribution of possible ${\overrightarrow{x}}_{i}$ values.

This average aggregation probability $F$ then determines the average likelihood for pairs of individuals to merge into a new clump at a given timestep $t$. $F$ has possible values ranging from 0 to 1. An $F$ value of 1 indicates any individual could fit into any group. Given a uniform initial composition, i.e. a uniform distribution of possible ${\overrightarrow{x}}_{i}$ values, an $F$ of 2/3 indicates that new members should be similar to existing members (i.e., homophily); an $F$ of 1/3 indicates a new member should be of a specific type that is currently under-represented in the group (i.e., heterophily). How the groups emerge and evolve depends on this collective chemistry embedded in $F$. As the model evolves in time, a finite non-negligible fraction of the total population can condense into a single large cluster—or equivalently, a giant connected component GCC in a network system (SI, Sect. 2.4–2.6). The expression for the time of the onset of the gel (i.e. appearance of the online group) is derived in SI Sect. 2.4. The evolution of the gel size is obtained by means of the exponential generating function and derived in SI, Sect. 2.6.

## Results

The political, social, and behavioral differences between the two movements are stark, yet we find many similarities at the system level in terms of the patterns their online growth seems to follow. Our mathematical theory (Sect. 2.2) of aggregation-with-heterogeneity (Fig. 3) makes specific predictions about the onset time and growth of the individual groups (Figs. 1, 2, using the data given in SI Fig. S5) and the overall movement (Fig. 5), and how these can be changed (Fig. 4):

1. It predicts that there is a single dynamical equation that governs the emergence and evolution of each individual group (Figs. 1, 2) and also the overall movement (Fig. 5A,B). This single equation is a generalized shockwave equation $\frac{\delta \upepsilon }{\delta t}=\frac{\delta \upepsilon }{\delta y}\frac{2F}{N}\left(\frac{\upepsilon }{N}-1\right)$, which is derived in SI Sect. 2 (specifically Eq. (16) where $F$ and $N$ measure the average aggregation probability and pool size for potential online recruits respectively, and the function $\upepsilon ={\sum }_{k=1}^{N}k{n}_{k}{e}^{yk}$ is known as a generating function (SI Sect. 2.6). While $\upepsilon$ doesn't represent any one physical variable in the system, it is instead a convenient sum from which physical values can be generated, like a partition function in statistical physics.
2. It predicts that the solution to this single shockwave equation corresponds to the size of each group in Figs. 1 and 2, and the overall movements in Fig. 5. Each is predicted to vary in time as $G\left(t\right)=N\left(1-W\left(\left[\frac{-2Ft}{N}\right]exp\left[\frac{-2Ft}{N}\right]\right)/\left[\frac{-2Ft}{N}\right]\right)$ where $W$ is the Lambert function and the appropriate values of $F$ and $N$ in each case are used. This is derived explicitly in SI Sect. 2.6 (Eq. (21)).
3. It predicts that each group in Figs. 1,2 and the overall movement in Fig. 5A,B, will have its own tipping point time ${t}_{onset}=\frac{N}{2F}$ which signals the onset of macroscopic growth. This tipping point is, in the limit of large $N$, a dynamical phase transition. It corresponds to the time at which the individual groups emerge in Fig. 1 for the Boogaloo groups, and in Fig. 2 for the ISIS groups, and where the overall movements emerge in Fig. 5A,B. Its value is shown on the horizontal axes in Figs. 3 and 4, in scaled form, for different values of $F$.
4. It predicts that the group size distribution at the onset ${t}_{onset}$ (see Fig. 5 insets) will be a power-law with a negative exponent of magnitude $5/2=2.5$.

These predictions are consistent with what we observe in the empirical data. Specifically, the onset times ${t}_{onset}$ and growth curves $G\left(t\right)$ for each Boogaloo group and ISIS group in Figs. 1 and 2, and for the movements as a whole in Fig. 5, are well-described by these mathematical predictions. Moreover, Fig. 5A, B insets show that the individual group sizes at ${t}_{onset}$ exhibit the predicted negative power-law exponent of magnitude $2.5$ (see SI Sect. 3 for analysis). Since the theoretical formulae are derived for very large $N$, the predicted onsets are too sharp: but at the expense of losing the closed-form formulae, we can extend the theory to account for finite $N$ as in the empirical data. The transition then becomes smooth like the empirical data, with the size at the onset varying as ${\left[N\right]}^{-1/3}$ as opposed to being strictly zero. This smoothing for smaller $N$ is shown explicitly in SI Fig. S4. We note that a larger $N$ is related to a sharper predicted onset because of an effect similar to a phase transition in physics: as $N$ increases, it becomes clearer when the largest component is a substantial fraction of the total population or not, whereas for small $N$ this is less clear and hence the transition is smoother. Because aggregation is happening in time, a sharper transition translates to a quicker change between no noticeable group and a noticeable group (i.e., no gel and a gel, or no giant connected component and a giant connected component). The closed-form formulae underlying predictions (1)–(4) above, become increasingly accurate as $N$ increases since they are calculated from coupled differential equations for the average numbers of clusters of a certain size (SI Sect. 2). They are less accurate when $N$ is small because fluctuations away from the average become larger relative to the average. This is akin to the law of large numbers and the central limit theorem. The equations for small $N$ can still be written down but they cannot be solved exactly. The theoretical results in Figs. 1, 2, 3, 4 and 5 correspond to the equations for large $N$ in predictions (1)–(4) above. On a related technical point, as $N$ becomes small, the onset time ${t}_{onset}=\frac{N}{2F}$ decreases but also becomes less accurate. The pathological limit of $N\to 0$ yielding ${t}_{onset}\to 0$ is simply a statement that for a few particles the gel will form almost instantaneously, e.g. in a population of $N=1$ a gel of size 1 already exists at the outset by definition.

The average aggregation probability $F$, which depends directly on the population heterogeneity (SI Sect. 2.2), is similar for each movement (Fig. 5A,B), with both $F$ values being statistically indistinguishable from $1/3=0.33,$ which is the value predicted mathematically for aggregation favoring diversity in a population with uniformly distributed $\left\{{\overrightarrow{x}}_{i}\right\}$ (see Discussion in SI at the end of Sect. 2.2). This would suggest that each movement develops by aggregating diverse sets of supporters from the global online user pool. Figure 1 inset shows that the membership heterogeneities $F$ of individual Boogaloo groups are also close to $1/3$, which suggests that individual Boogaloo group formation is also driven by the same preference for diversity as the entire Boogaloo movement. This is consistent with the eclectic mix of memes and ideas we observe in the content of each Boogaloo group, and the lack of any increase in topic coherence that we observe from our dynamic Latent Dirichlet Allocation topic analysis of their narratives (see SI Sect. 5). By contrast, individual ISIS groups have $F$ values closer to $2/3$ (Fig. 2 inset), which suggests that once inside the ISIS movement, supporters form into groups that are each internally homogenous and have a well-defined narrative. Overall, this suggests that while Boogaloo and ISIS recruits join the overall movements driven by diversity, Boogaloos continue with this diversity driver when forming and joining an individual group, while ISIS supporters prefer a group to have a single narrative.

## Discussion and conclusions

By incorporating the interplay between individual human heterogeneity and group formation^32^ online, our mathematical theory has placed these extremist movements' evolutions on a similar footing—akin to a single equation in physics explaining the different trajectories of different objects. While there are important individual-level and movement-level characteristics that affect the growth of these groups, our identification of a system-level mathematical order^33–37^ opens the door to a common set of mitigation strategies. Specifically, our mathematical theory suggests that social media platforms can mitigate the growth of new forms of online extremism by nudging the collective chemistry of online movements, specifically by changing the average aggregation probability $F$ as shown in Fig. 4. Online extremist groups can show remarkably quick growth and adaptation, particularly those focused around fresh narratives as in Figs. 1 and 2, and react quickly when they realize their content is being moderated. By contrast, long-standing online communities typically change slowly over time (SI Fig. S6). But while sweeping shutdowns of online groups are sometimes called for, this tactic has the disadvantage of being highly visible (and thus sometimes provoking and energizing extremists), and also can be circumvented when individuals move to unmoderated platforms. The nudging tactics we suggest below may have the benefit of significantly slowing these groups while being less visible and thus less likely to spur rapid adaptation.

We start by recalling that groups with smaller $F$ grow more slowly, and even slightly decreasing $F$ at the level of the entire movement or individual group delays the onset (since ${t}_{onset}\propto 1/F)$ and flattens the growth curve $G\left(t\right)$: specifically, $-\Delta F/F =\Delta {t}_{onset}/{t}_{onset}$. Figure 4 then shows explicitly how by changing the $F$ value, a delaying of the onset and flattening of the curve can be achieved. The discussion in SI Sect. 2.2 shows quantitatively how such specific $F$ values could be tailored. Social media platforms can use several tactics to perform this nudging of the $F$ value. One example is by injecting extremists’ online spaces (e.g., Facebook Page) with topically diverse material, such as by posting ads and banners that present content about which members of the group are likely to disagree.

Platforms can also nudge the composition of the overall pool of potential recruits to these movements. Platforms already use algorithms to provide users with recommendations of groups to join, including extremist groups to the extent platforms’ algorithms predict users would be interested in such groups. By altering such algorithms, i.e., by suggesting or recommending the target group to members of a heterogeneous set of other groups, a platform can nudge a more heterogeneous set of individuals to join the target group. Such tactics bias the interaction of dissimilar individuals such that aggregation favors dissimilar ${\overrightarrow{x}}_{i}$’s, thus lowering $F$. Such tactics may be more effective with respect to smaller (and hence potentially less robust) groups, such that the power-law distribution at the onset can be disrupted (see SI), which in turn disrupts the dynamical phase transition and delays the onset of support. We note the important caveat that the suggested interventions of our model are theoretical and have not yet been tested. At the same time, it is important to be able, within the bounds of a model, to make predictions that can guide discussions and may be testable in a limited setting. As with all such policies, they would first need to be fully tested in a controlled environment.

Explaining *why* the Boogaloos have suddenly emerged requires deeper social, political, and economic debate, part of a much broader debate that is beyond the scope of this paper. However, we can hint at a possible mathematical description by extending the heterogeneity-driven aggregation toward a generalized version of the sociological Seceder Model of Dittrich et al. and Halpin-Healy et al.^48–50^. This model has a more sophisticated rule for aggregation than the one used so far in this paper, as follows. Following the description of Halpin-Healy, the model considers a population of *N* individuals, each having a *d*-dimensional opinion vector which represents some ideological or political position. One member of the population is chosen at random to revise their position. This person picks *m* other individuals, who will help the individual form an opinion and hence change the individual's opinion vector. From this selection multiplet of size *m*, the individual chooses the most distinct member, meaning the member farthest from the average. The individual then chooses a new value of the *d*-dimensional opinion vector, near that farthest person's value. In this way, the model captures two opposing tendencies: conformity and dissent. Specifically, if the group is initially tightly-knit, the model's mechanism acts to enhance homogeneity since the individual, who could originally be quite far from that subset in ideological space, leaves that position and effectively conforms. By contrast, if there is an outlier among the selection multiplet, the individual chooses that outlier as opposed to the mainstream view.

**Figure 6:**
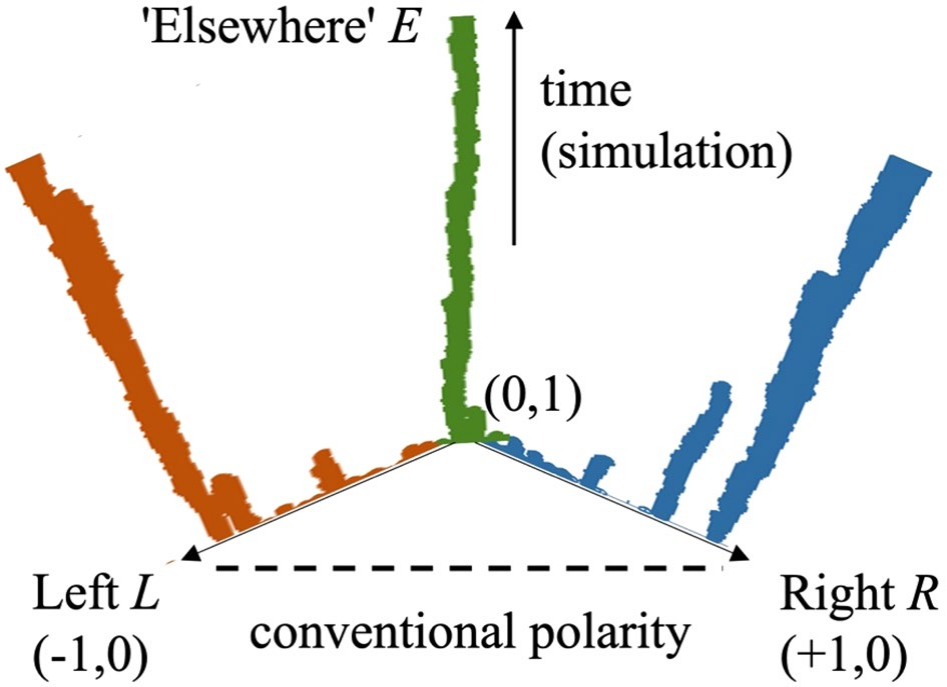
Computer simulation of the Seceder mechanism of Dittrich et al. and Halpin-Healy et al.^48–50^. Though a similar finding of emergent branches appears for more general dimension choices, for illustrative purposes we generated this output by splitting the simple one-dimensional output into values from 0 to 0.5 to provide one axis (branches and clustering shown in orange) and 0.5 to 1 to provide another (branches and clustering shown in blue) hence the triangular shape for the x axis. The green branch mimics the Boogaloo movement and classifies as ‘elsewhere’ since it is neither on the left or right of the one-dimensional axis. Each colored dot represents the position of an agent in the system at a particular time and with a particular value along the one-dimensional line. The branches emerge as a result of the model’s competition between the pressure to conform and the desire to dissent. The plot shows the aggregation of agents around specific values, so the wider the line, the more agents with similar values in that branch. Since the vertical axis is time in the computer simulation, each branch generates a tall structure with height equal to the time. Since the sizes of these branches of individuals fluctuate as time progresses, these tall structures have non-constant width.

The results of this Seceder Model are shown in Fig. 6 while SI Sect. 4 has full mathematical details. The Seceder Model’s competition between the pressure to conform and the desire to dissent, means that distinct individuals can generate a following. This may have been the case with the Boogaloos, who are neither consistently far-right nor far-left and instead lie in another dimension in ideological space as suggested by Fig. 6. Such a competition is consistent with the Boogaloos’ eclectic mix of fads (e.g., memes) and fashions and the lack of any increasing topic coherence in their narratives (see SI Sect. 5). Even for a one-dimensional opinion vector ($d=1$) the results of this model already show the emergence of a third movement, in addition to far-left and far-right, that mimics the emergence of the Boogaloos. Specifically, the Seceder model predicts the emergence of 3 stable movements (Fig. 6) and it could in the future be used to estimate the number of new extremist ‘branches’ to eventually expect. We leave this exploration for future work. To back up our claims about the nature of the Boogaloo groups’ narratives being diffuse and not clearly far-right or far-left, the SI Sect. 5 details results we have obtained using machine learning analysis of their groups’ narrative content. Specifically, the SI Fig. S7 shows that the topic coherence of the Boogaloo groups’ content tends to either decrease in time or stay roughly constant. It does not show any systematic increase.

One potential limitation of our study is that our mathematical analysis is intended for large numbers of potential recruits $N$ and the theoretical predictions are therefore not perfect. While the smooth onset of the empirical growth curves can be reproduced at the expense of a loss of closed-form formulae as discussed earlier, there are still unexplained bumps and jumps. However, these can also be reproduced if we allow for an online influx of potential recruits and $N$ then becomes a function of time. We also need to study future extremist movements as they emerge online over time, to check the further generality of our findings. However, the Boogaloos and ISIS are certainly movements of high current salience, and the SI shows that their hidden mathematical order, as reported here, does not arise for other online human aggregation behaviors (see SI Sect. 3.2). We therefore hope this mathematical system-level analysis is seen as a useful complement to research using other tools and levels of analysis conducted by social scientists and others.

While our study is a comparative one of two very different movements, within each movement we analyze a large number of groups that include vast numbers of users. The groups shown in each case in Figs. 1 and 2 are a small subset presented for visual clarity, and the full lists of groups we analyze, and their results, are provided in the SI (SI, Sect. 3.2). If and when a comparable movement emerges again, the theory offers that predictions that could be further tested against it.

In conclusion, our results represent a step toward an eventual system-level understanding of the emergence of extremist movements. We have compared the growth of the Boogaloos, a new and increasingly prominent U.S. extremist movement, to the growth of ISIS, a militant, terrorist organization based in the Middle East that follows a radical version of Islam. We have given evidence that the early dynamics of these two online movements follow the same mathematical order despite their stark ideological, geographical, and cultural differences. The mathematical material that we developed, which is given in detail in the SI, builds on work in the physics, chemistry, and mathematics literature, with the generalization that particles (individuals) that are typically treated as identical now have individual heterogeneity. Our findings suggest policies to address online extremism and radicalization—for example, showing how actions by social media platforms can disrupt the onset and ‘flatten the curve’ of such online extremism by nudging its collective chemistry. We stress the important caveat that the suggested interventions of our model are theoretical and have not yet been tested. As with all such policies, they now need to be fully tested in a controlled environment. At the same time, they do provide a new quantitative platform to facilitate progress in countering online extremism.

## Supplementary Information

Supplementary Information.
