# Supplementary material for: Hidden order across online extremist movements can be disrupted by nudging collective chemistry
Source: Sci Rep. 2021 May 19;11:9965. doi: 10.1038/s41598-021-89349-3 (PMC8134557; doi:10.1038/s41598-021-89349-3)
Supplement: Supplementary file 1 — Supplementary Information. [file 41598_2021_89349_MOESM1_ESM.pdf]

Supplementary Information (SI)

1. Data Collection

2. Details of the Generalized Aggregation Theory in Figs. 1-5

3. Data Analysis for Figs. 1, 2 and 5

4. Details of Seceder Model in Fig. 6

5. Boogaloos' Topic Coherence in Time

1. Data Collection

As described in the main paper, we collected daily, public data at the community level (i.e. Facebook Pages for Boogaloos, VKontakte Groups for ISIS) following our earlier methodology in Refs. 17-19 of the main paper. Such communities are known to facilitate coordination and play a greater role in nurturing narratives than platforms like Twitter which have no pre-built community tool and are instead designed for broadcasting short messages. The Boogaloos do not have a clear partisan base, and often ridicule leaders of both major U.S. parties (i.e. they are 'elsewhere' as in Fig. 6 of the main paper). They have no formal institutional structure or hierarchy, but instead organize loosely on numerous social media pages with an eclectic mix of fads and fashions. ISIS, by contrast, adheres to a completely distinct ideology (a radicalized form of fundamentalist Islam), it was initially organized offline and later used social media to gain followers, it has a formal leadership and hierarchy, it is based in the Middle East rather than the U.S., and it seeks to establish itself as a formal state with authority over all Muslims.

2. Generalized Aggregation Theory in Figs. 1-5

Here we present the mathematical details of the group formation discussed in the main paper. It involves the online dynamics of clumps of individuals that may then aggregate into an online gel. These clumps are equivalent to small pieces of a network that are disconnected from the rest of the network, or pieces that are weakly connected to the rest of the network, since both can be described by the same approximate mean-field equations that we develop. These lead to a gel forming, or equivalently the giant connected component (GCC) of the network, which means it is a macroscopically large cluster. In Figs. 1-4, this gel or GCC is an individual group supporting ISIS or Boogaloos, while in Fig. 5 it is the entire movement, Both are valid uses of the mathematics, just at different scales.

Before proceeding, we comment on the potentially confusing terminology clump, cluster, community and group. Mathematically, there is no confusion since the issue simply concerns whether a collection of objects is microscopic (i.e. a lot less than the size of the population and hence not scaling with the size of the population) or macroscopic (i.e. a finite fraction of the population and hence scaling with the size of the population). Prior to the dynamical phase transition to a gel (or equivalently GCC), all clumps of objects are much smaller than the relevant population size and are hence microscopic at that scale – i.e. for Figs. 1-4, the clump being much smaller than the size of the relevant gel or GCC means it is much smaller than an individual Boogaloo or ISIS group, while in Fig. 5 the clump being much smaller than the than the size of the relevant gel or GCC means it is much smaller than the overall Boogaloo or ISIS movement. It is just a change in language according to scale, but the mathematics is exactly the same. In the case that the gel is considered the movement as a whole (Fig. 5), each clump is one of the Facebook Pages (for Boogaloos) or VKontakte Groups (for ISIS) and hence is a cluster or is like a 'community' in the terminology of social media. These form a power-law distribution of sizes with exponent 5/2 at the onset of the dynamical phase transition and hence at the point that the gel (or equivalently the GCC)

emerges, and at this stage no single cluster has a size comparable to the population. In the case of Figs. 1 and 2 that the gel is a single Facebook Page (for Boogaloos) or VKontakte Group (for ISIS), the clumps are microsopically small clumps of correlated individuals (e.g. from WhatsApp) who come together to form a single Page or Group, and these clumps are not observable to us in our data collection. In principle, they also form a power-law distribution of sizes with exponent 5/2, but we do not have that level of granularity of data and so we do not study it further here. Under aggregation, following the dynamical phase transition, a clump becomes so large that it is macroscopic, i.e. a single large clump emerges that has a size comparable to the population size. This large clump is the gel, or a GCC in a network setting. In the end, the mathematical equations are exact and precise in terms of their form, while words such as 'group' can suffer from vagueness and alternative interpretations.

The key takeaway is that the mathematical aggregation theory that we present here to produce the results in Figs. 1-5, can equivalently be viewed as applying to the linking together of objects in a network, or to the aggregation of objects in a more abstract setting (see Figs. S1-S3) since the coupled equations are equivalent in both cases at the mean-field level – as shown in the simpler case of identical objects in the book of Redner [1] – and it can be applied at different scales as in Fig. 1-4 for a single group, or Fig. 5 for an overall movement. The words chosen to describe what these clumps, gels etc. are, then become a matter of choice and will be nuanced by academic discipline.

2.1 Same Mathematics Applied at Two Different Scales: Figs. 1-4 and Fig. 5

We apply this aggregation theory at 2 different scales in the main paper: (1) the emergence of the *overall* movement is a gel (or equivalently GCC in a network) forming from the background pool of users on the Internet as in Fig. 5 of the main paper; and (2) the emergence of a *single* Facebook Page (for Boogaloos) or VKontakte Group (for ISIS) is a gel (or equivalently GCC in a network) that forms from within the movement itself as in Figs. 1 and 2 of the main paper. We could also add fragmentation to represent the later shutting down of links by moderator action etc., however we restrict our focus here to the aggregation process through which the movement is growing. In the language of networks, we note that the individual heterogeneity that we incorporate is an intrinsic heterogeneity of an individual node, i.e. it is not a node's degree of connectivity but rather an intrinsic property of that node. More generally, the gel (or equivalently the GCC in a network) can emerge from some coupled combination of the axes corresponding to each dimension of the internal 'character' variable described below, however the same derivation applies.

2.2 Individual Heterogeneity and Mean-Field Aggregation Probability F

We include heterogeneity among the interacting individuals, and consider the situation where this heterogeneity helps dictate the evolution of the aggregation process, i.e. it ultimately produces the distinct flavors of the movement as a whole, or of the individual

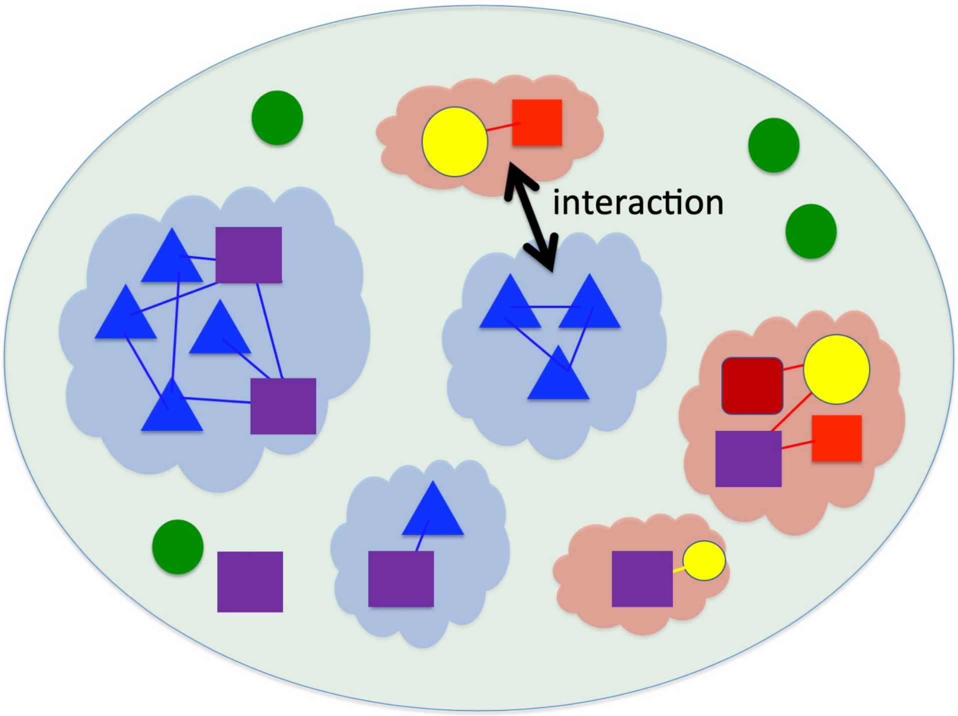

Figure S1: Overview of our model of the system of interest. It comprises a population of interacting, heterogeneous individuals (i.e. objects/nodes) and can be represented as a partially connected network of nodes, or in a more abstract way as pockets of coupled or correlated entities. The gel – or equivalently the giant connected component GCC – can then emerge from these as a result of aggregation.

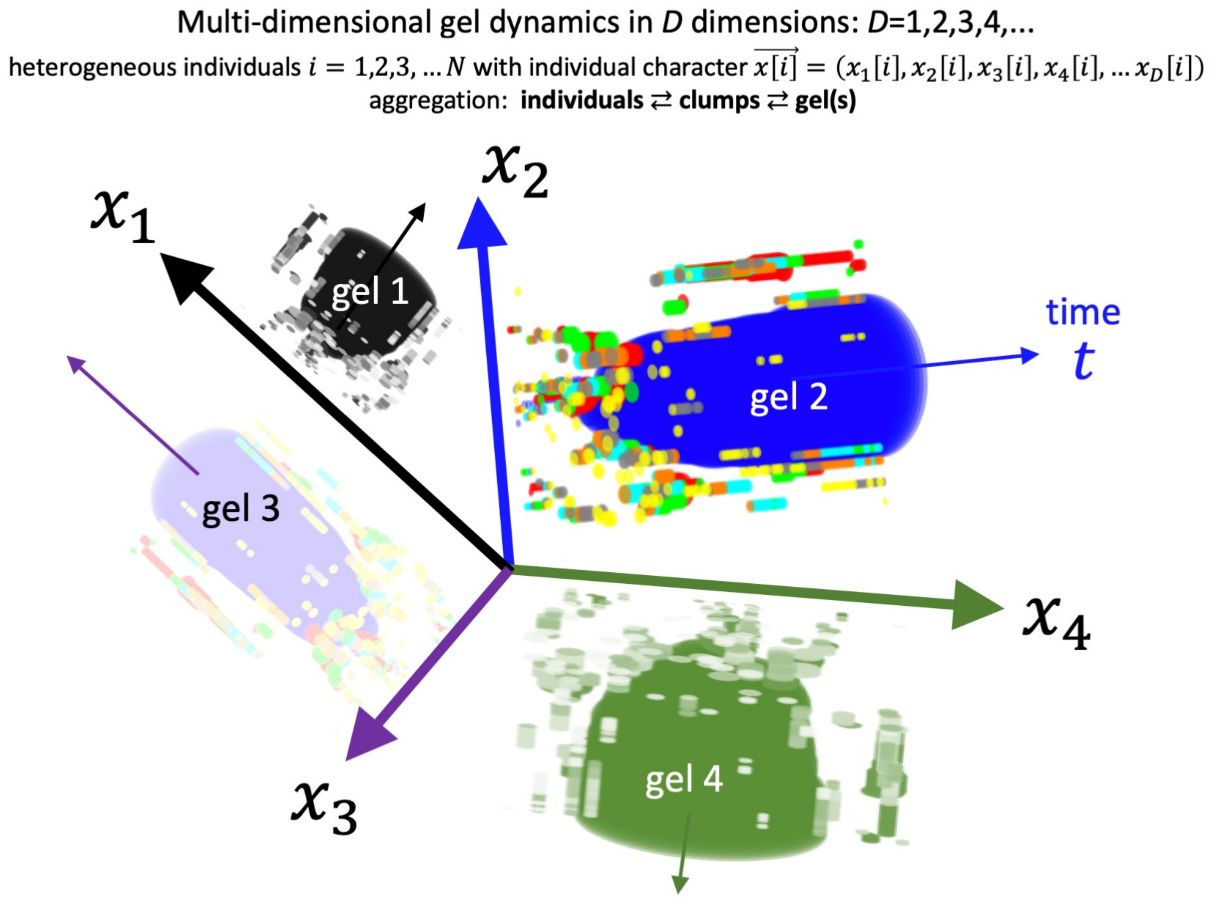

Figure S2: Structure of our model. A gel – or equivalently a giant connected component GCC in a network – can emerge along any of the character dimensions. More generally, gels could form that combine character dimensions, and essentially the same mathematics applies since this only affects F at the mean-field level, but we keep the story simpler for the purposes of this discussion. We apply this aggregation theory at 2 different scales in the main paper: (1) the emergence of the *overall* movement is a gel forming from the background pool of users on the Internet as in Fig. 5 of the main paper; and (2) the emergence of a *single* Facebook Page (for Boogaloos) or VKontakte Group (for ISIS) is a gel that forms from within the movement itself as in Figs. 1 and 2 of the main paper.

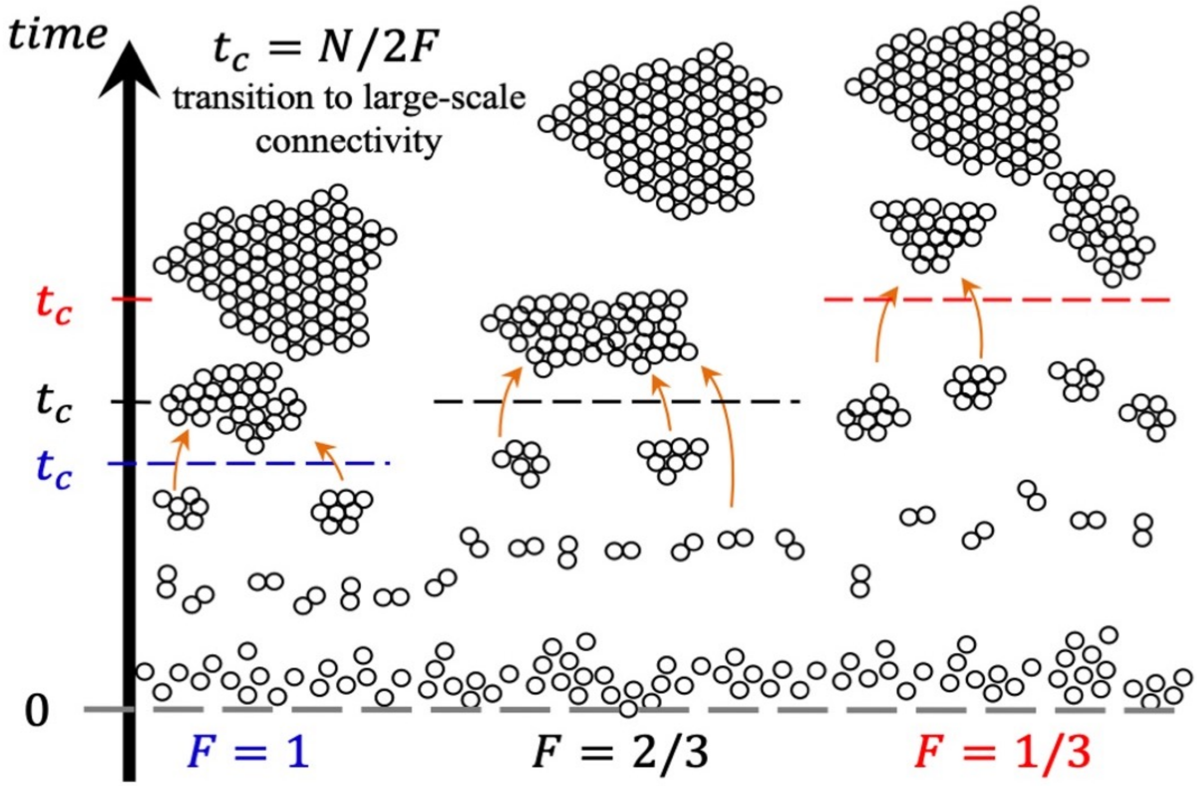

Figure S3: The dynamical phase transition to a gel at time t_{onset} for the simple case of a one-dimensional (i.e. scalar) individual character x for different character-dependent aggregation mechanisms and hence different F values as shown in Figs. 3,4 of the main paper.

Facebook Pages seen in the Boogaloo (or VKontakte Groups for ISIS) ecology online. A hidden variable x that we for simplicity call 'character', is randomly assigned to each individual taken from a given distribution $q(x)$. While this is undoubtedly a crude way of adding individual human heterogeneity, it is common practice in computational social science: moreover, x could be made a general D-dimensional vector $\vec{x} = (x_1, x_2, \ldots x_D)$ where D is an arbitrarily large number, without changing the analysis. A gel can hence form along each of these D dimensions, or combination of them, yielding a set of $\leq D$ gels, i.e. a set of $\leq D$ Boogaloo Facebook Pages or a set of $\leq D$ ISIS VKontakte Groups as observed empirically in Figs. 1 and 2. Also, x could be made time-dependent at the cost of analytical complexity. The interaction between individuals is described in terms of their similarity or dissimilarity (diversity) and hence is some function of their respective x values.

We incorporate this by first defining the similarity S_{ij} between individual i and individual j as $S_{ij} = 1 - |x_i - x_j|$, so that individuals with alike character have a high similarity and otherwise for a pair of individuals with unlike character. We consider that the probability of aggregation for any two individuals i and j is given by $\mathcal{C} = S_{ij}$. Our definition also recognizes the opposite mechanism of diversity (i.e. dissimilarity) which tends to form clumps of dissimilar individuals, where the aggregation probability between i and j is $\mathcal{C} = 1 - S_{ij}$. The random case is recovered in the limit where the aggregation probability is independent of x, which is $\mathcal{C} = 1$. Doing this in $D = 1, 2, 3...$ etc. dimensions leads to a set of diverse flavors of gel emerging, as observed empirically. The heterogeneous aspect of the aggregation process is then transferred to the equations for the evolving population by means of a mean-field probability for aggregation. For example, for a uniform character distribution $q(x)$, the probability density function (PDF) of the similarity $y = S_{ij}$, for aggregation through similarity (homophily) is $f(y) = 2y$ and hence the mean-field aggregation probability F, becomes:

$$F = \int_0^1 y f(y) dy = 2/3. \tag{1}$$

By contrast, for aggregation through dissimilarity (heterophily) defining $z = 1 - S_{ij}$, the PDF $f(z) = 2(1 - z)$ resulting into a mean-field aggregation probability F, of:

$$F = \int_0^1 z f(z) dz = 1/3. \tag{2}$$

Different calculations and values for F will follow according to the choice made for the initial composition of the population $q(x)$, and also for variants away from pure homophily and pure heterophily. In all cases, the resulting mean-field probability F determines the likelihood for any pair of individuals i and j to merge into a new clump at a given timestep t. For example, (1) a delta-function for the character distribution $q(x)$ which can be peaked at any value, gives $F = 1$ for homophily, $F = 0$ for heterophily, and $F = 1$ for character-independent aggregation; (2) a bimodal function for the character distribution $q(x)$ peaked symmetrically at 0 and 1, gives $F = 1/2$ for homophily, $F = 1/2$ for heterophily, and $F = 1$ for character-independent aggregation; the above result (3) a uniform function for the character distribution $q(x)$ (i.e. flat) gives $F = 2/3$ for homophily, $F = 1/3$ for heterophily, and $F = 1$ for character-independent aggregation.

2.3 Coupled Differential Equations for Aggregation Dynamics

We start by developing a set of rate equations for the number of small clumps of individuals of size k ($k = 1, 2, ...$) over time. The mathematical material that we present here builds on from work in the physics, chemistry and mathematics literature, with the generalization that particles (individuals) that are typically treated as identical now have individual heterogeneity.

Aggregation theories describe the interaction and growth of clumps traditionally by means of kernels that depend on the sizes of the interacting clumps. That is, two clumps of sizes i and j merge forming a new clump of size $i + j$ at a rate given by the kernel K_{ij}. When the aggregation rate increases sufficiently rapidly with the size of the clumps, the system experiences a large-scale transition where a non-negligible fraction of the total population gather into the largest clump (i.e. a gel or equivalently a GCC forms). The product kernel interaction, which can be generalized, has been empirically verified for communities in human communication and collaboration networks [3]. Its distance independence reflects the global reach of online interactions, and serves as a mean-field approximation in other settings. Specifically, Palla et. al. showed empirically [3] that human grouping phenomena follow closely the traditional aggregation theory for the case in which the kernel is proportional to the sizes of the interacting clumps, i.e., $K_{ij} \propto ij$. With this in mind we can rewrite a set of equations for the number of clumps of size k (n_k), for the heterogeneous system as:

$$\dot{n}_k(t) = -2F\frac{kn_k}{N^2}\sum_{r=1}^{\infty} rn_r + \frac{F}{N^2}\sum_{r=1}^{k} rn_r(k-r)n_{k-r}, \quad k \geq 2 \tag{3}$$

$$\dot{n}_1(t) = -2F\frac{n_1}{N^2}\sum_{r=1}^{\infty} rn_r, \quad k = 1, \tag{4}$$

where N is the subpopulation from which a particular flavor of future Facebook Page or VKontakte Group (gel) might emerge in Fig. 1 if gelation occurs. The first term of Eqs. (3) and (4) represents the population of clumps of size k that merge with other clumps, while the second term in Eq. (3) is the population of smaller clumps that merge to form clumps of size k, consisting of the well-known product kernel. By considering $N = \sum_{r=1}^{\infty} kn_k$, Eq. (4) can be immediately solved and the expression for the number of isolated individuals is:

$$n_1(t) = Ne^{-\frac{2F}{N}t}, \tag{5}$$

where we have assumed that initially the system is comprised by individuals only, ($n_1(0) = N$). Equation (5) can be used to solve equation (3) for the case of $k = 2$. The result is:

$$n_2(t) = Fte^{-\frac{4Ft}{N}}. \tag{6}$$

Similarly, the found expressions for n_1 and n_2 are used in equation (3) to solve for $k = 3$ (n_3) resulting in:

$$n_3(t) = \frac{2F^2t^2}{N}e^{-\frac{6Ft}{N}}. \tag{7}$$

This recursive process is repeated to solve for higher k values. The general expression for any $k \geq 2$ is found to be:

$$n_k(t) = \frac{1}{k!}\left(\frac{k}{N}\right)^{k-2} (2Ft)^{k-1} e^{-\frac{2kF}{N}t}. \tag{8}$$

2.4 Onset Time of Gel or equivalently Giant Connected Component (GCC)

At some later point into the dynamics, a finite non-negligible fraction of the total population may condense into a single large cluster called a 'gel', or equivalently, a giant connected component GCC in a network system. This phenomena is known as gelation and divides the dynamics of the system. In short, in the case of Figs. 1 and 2, each Facebook Page (for Boogaloos) or VKontakte Group (for ISIS) is a large cluster which we can call a gel. After the gel is formed, the moments of the size distribution become decomposed into the small clumps (or *solution*) and the gel in the following way:

$$M_j = \sum_{k\geq 1} k^j n_k = \sum_{sol} k^j n_k + (k^j n_k)_{gel}. \tag{9}$$

The importance of this decomposition becomes evident when analyzing the zeroth moment, $M_0 = \sum_{k\geq 1} n_k$, which provides the number of clumps of any size. By looking at its first derivative we find:

$$\begin{aligned}
\frac{dM_0}{dt} &= \sum_{k\geq 1} \frac{dn_k}{dt} \\
&= -2F\sum_k \frac{kn_k}{N} + \frac{F}{N^2}\sum_k \sum_{i+j}^{k} (in_i)(jn_j) \\
&= -F, \quad \Rightarrow \quad M_0(t) = N - Ft. \qquad (10)
\end{aligned}$$

The solution for the zeroth moment becomes negative when $t > N/F$ which is problematic since M_0 gives the total number of clumps present. This problem is solved by using, above the gel point, $\sum_{k\geq 1} kn_k = N - G$, where G is the size of the gel. With this correction the derivative of the zeroth moment becomes:

$$\begin{aligned}
\frac{dM_0}{dt} &= -\frac{2F}{N}(N-G) + \frac{F}{N^2}(N-G)^2 \\
&= \frac{F}{N}(N-G)\left(\frac{N-G-2N}{N}\right) = -\frac{F}{N^2}(N-G)(N+G) \\
&= \frac{F}{N^2}\left(G^2 - N^2\right). \qquad (11)
\end{aligned}$$

Note that equation (11) indicates that the number of clumps stops decreasing when the gel reaches the system size N. The appearance of the gel is mathematically manifested as

a singularity in the second moment of the size distribution. The evolution of the second moment is given by:

$$\frac{dM_2}{dt} = \sum_{k\geq 1} k^2 \frac{dn_k}{dt}. \tag{12}$$

Using equation (3), we can work out this expression as follows:

$$\begin{aligned}
\frac{dM_2}{dt} &= \sum_{k\geq 1} \left(\frac{k}{N}\right)^2 F \sum_{i+j=k} in_i jn_j - \sum_{k\geq 1} k^3 n_k \frac{2F}{N} \\
&= \sum_{\substack{i\geq 1\\ j\geq 1}} (i+j)^2 \frac{F}{N^2} in_i jn_j - \sum_{k\geq 1} k^3 n_k \frac{2F}{N} \\
&= \sum_{\substack{i\geq 1\\ j\geq 1}} \left(i^3 jn_i n_j + 2i^2 j^2 n_i n_j + ij^3 n_i n_j\right) \frac{F}{N^2} - \sum_{k\geq 1} k^3 n_k \frac{2F}{N} \\
&= \sum_{\substack{i\geq 1\\ j\geq 1}} \left(i^2 n_i\right)\left(j^2 n_j\right) \frac{2F}{N^2} + \left(i^3 n_i jn_j + in_i j^3 n_j\right) \frac{F}{N^2} - \sum_{k\geq 1} k^3 n_k \frac{2F}{N} \\
&= \sum_{\substack{i\geq 1\\ j\geq 1}} \left(i^2 n_i\right)\left(j^2 n_j\right) \frac{2F}{N^2} + \sum_{k\geq 1} k^3 n_k \frac{2F}{N} - \sum_{k\geq 1} k^3 n_k \frac{2F}{N} \\
&= \sum_{\substack{i\geq 1\\ j\geq 1}} \left(i^2 n_i\right)\left(j^2 n_j\right) \frac{2F}{N^2} \\
&= M_2^2 \frac{2F}{N},
\end{aligned} \tag{13}$$

which gives a closed differential equation for the second moment of the size distribution. The solution for the initial condition where all clumps are of size one ($M_2(0) = N$), is:

$$M_2(t) = \left(\frac{1}{N} - \frac{2Ft}{N^2}\right)^{-1}, \tag{14}$$

which has a singularity at the time

$$t_{onset} = N/2F \tag{15}$$

which is the onset time at which the transition to having a gel takes place.

2.5 Different Onset times for Gel or GCC in Figs. 1-5

This critical time for the onset of the gel or equivalently GCC, $t_{onset} = N/2F$, depends on (a) the mean-field aggregation probability F and hence on the nature of the aggregation process as well as the initial character distribution of the population $q(x)$, (b) the size of

pool of potential recruits online N. For a uniform character distribution, unlike clumps (dissimilarity, or diversity) are slower to be formed and hence the transition occurs at a later time than alike cluster formation (homophily). Random clumps are the quickest to form since they have the maximum mean-field aggregation probability per timestep ($F = 1$). We recall from above, the examples of (1) a delta-function for the character distribution $q(x)$ which can be peaked at any value, gives $F = 1$ for homophily, $F = 0$ for heterophily, and $F = 1$ for character-independent aggregation; (2) a bimodal function for the character distribution $q(x)$ peaked symmetrically at 0 and 1, gives $F = 1/2$ for homophily, $F = 1/2$ for heterophily, and $F = 1$ for character-independent aggregation; the above result (3) a uniform function for the character distribution $q(x)$ (i.e. flat) gives $F = 2/3$ for homophily, $F = 1/3$ for heterophily, and $F = 1$ for character-independent aggregation. These different F values together with different values of the pool N, explain the different onset times shown in Figs. 1-5.

2.6 Size of Gel or equivalently Giant Connected Component (GCC)

The expression for the evolution of the gel size is obtained by means of the exponential generating function $\mathcal{E}(y,t) \equiv \sum_{k\geq 1} kn_k e^{yk}$. Hence:

$$\begin{aligned}
\frac{\partial \mathcal{E}}{\partial t} &= \sum_{k\geq 1} k \frac{\partial n_k}{\partial t} e^{yk} \\
&= -\frac{2F}{N}\sum_{k} k^2 n_k e^{yk} + \frac{F}{N^2}\sum_{i\geq 1}\sum_{j\geq 1}(i+j) i n_i j n_j e^{yk} \\
&= -\frac{2F}{N}\sum_{k} k^2 n_k e^{yk} + \frac{F}{N^2}\sum_{i} i^2 n_i e^{yi}\sum_{j} j n_j e^{yj} + \frac{F}{N^2}\sum_{i} i n_i e^{yi}\sum_{j} j^2 n_j e^{yj} \\
&= \frac{2F}{N^2}\mathcal{E}\frac{\partial \mathcal{E}}{\partial y} - \frac{2F}{N}\frac{\partial \mathcal{E}}{\partial y} \\
&= \frac{\partial \mathcal{E}}{\partial y}\frac{2F}{N}\left(\frac{\mathcal{E}}{N} - 1\right).
\end{aligned} \tag{16}$$

Equation (16) is known as the inviscid Burgers equation which is the simplest nonlinear hyperbolic equation and can be solved by the method of characteristics. For this type of partial differential equation, the characteristics are straight lines in the y-t plane where $\mathcal{E}$ is constant and have slope $\alpha(1-\mathcal{E}')$, where for simplicity we have defined $\alpha = 2F/N$ and $\mathcal{E}' = \mathcal{E}/N$. The equation of motion for y along the characteristic is therefore:

$$\frac{dy}{dt} = \alpha(1-\mathcal{E}'). \tag{17}$$

Since $\mathcal{E}$ (and hence $\mathcal{E}'$) is constant, the solution for $y(t)$ along the characteristic is:

$$y(t) = \alpha(1-\mathcal{E}')t + f(\mathcal{E}), \tag{18}$$

where $f(\mathcal{E})$ depends on the initial conditions which for the generating function we find it to be $\mathcal{E}(y, t=0) = Ne^y$ which yield $y(t=0) = \ln \mathcal{E}'$. The derivation moves forward as follows:

$$\begin{aligned} y &= \ln \mathcal{E}' + \alpha t(1-\mathcal{E}) \\ e^y &= \mathcal{E}' e^{\alpha t(1-\mathcal{E}')} \\ e^{y-\alpha t} &= \mathcal{E}' e^{-\alpha t \mathcal{E}'}. \end{aligned} \tag{19}$$

Now note that the generating function for $y = 0$ yields $\mathcal{E}(0, t) = N - G$ above the gel point and the following expression for the largest clump (cluster, i.e. gel or equivalently GCC) is found:

$$\frac{G}{N} = 1 - e^{-\frac{2Ft}{N^2} G}. \tag{20}$$

The solution of equation (19) can by written by means of the W-Lambert function as:

$$G = N(1 - W(ze^z)/z), \quad z = -2Ft/N. \tag{21}$$

which is the equation given in the main paper.

2.7 The 5/2 Power Law Distribution for Cluster Sizes

This theoretical model predicts an approximate power-law (PL) size distribution at the transition point with negative exponent of magnitude exactly $5/2 = 2.5$. This can be seen in the equation for $n_k(t)$ when we look at large k. Using the Stirling approximation this equation can be written as:

$$\begin{aligned} n_k(t) &\approx \left(\frac{e}{k}\right)^k \left(\frac{k}{N}\right)^{k-2} \frac{1}{\sqrt{2\pi k}} (2Ft)^{k-1} e^{-2kFt/N} \\ &= \frac{N^2}{2Ft} \left(\frac{2Ft}{N}\right)^k \frac{1}{\sqrt{2\pi}} e^{-\frac{2F}{N}k\left(t - \frac{N}{2F}\right)} k^{-5/2} \\ &= \frac{N}{\sqrt{2\pi}} \left(\frac{t}{t_{onset}}\right)^{k-1} e^{-\frac{k}{t_{onset}}(t - t_{onset})} k^{-5/2} \end{aligned} \tag{22}$$

which around $t \approx t_{onset}$, we can approximate $e^{-k(\tau - \ln\tau - 1)} \to e^{-k(1-\tau)^2/2}$, which yields:

$$n_k(t) \to \frac{N}{\sqrt{2\pi}} e^{-\frac{k}{2}(1 - \frac{t}{t_{onset}})^2} k^{-5/2}. \tag{23}$$

For $t = t_{onset}$ it yields a PL distribution with negative exponent of magnitude exactly $5/2 = 2.5$ that also serves as a signal of the gel transition. Traditionally, gelation refers to the sudden appearance of a macroscopic clump (macroscopic 'cluster' in the physics and chemistry literature) during the process of polymerization in chemical compounds [9, 10].

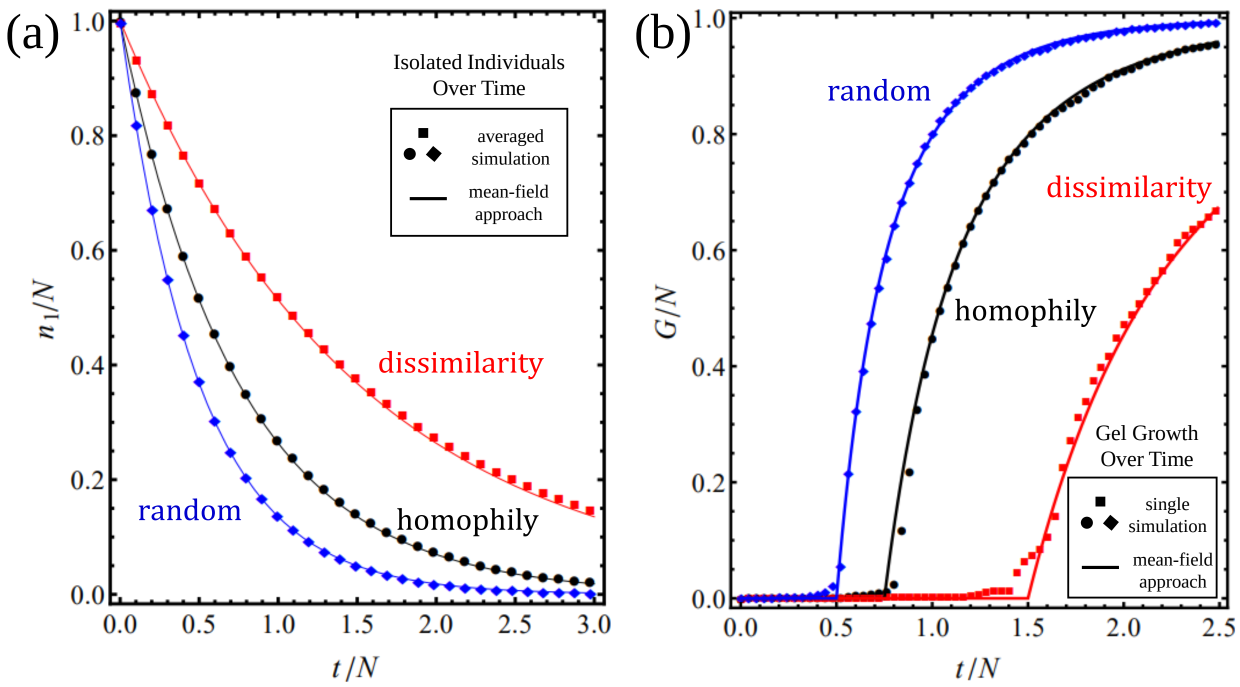

Figure S4: Evidence of the accuracy of our mathematical expressions, by comparing with stochastic computer simulations. (a) Evolution of the fraction of isolated individuals from theory and averaged stochastic simulations (500 realizations). (b) Size of gel from theory with single stochastic simulations. Each panel contains the results from the mechanisms homophily (i.e. similarity, kinship), heterophily (i.e. diversity, dissimilarity) and random. System size: $N = 10^4$.

The percolation model, in turn, has been one of the means used to understand the phenomenon of gelation but also to study connectivity transitions in different network architectures [11, 12]. For the particular case of random percolation [13, 14], the mean-field theory presented above provides the time-evolution of the clump size distribution [1]. Given that the mean-field theory describes the formation of clusters, one could argue it resembles more closely a gelation process than a network formation since details of the network architecture are not included beyond mean-field. But it is correct to mean-field level for both.

3. Data Analysis for Figs. 1, 2 and 5

We obtained data for ISIS support on the prominent platform VKontakte [2]. This site up to January of 2017 had 410 million users world-wide and is known to have been used to spread ISIS propaganda through online groups among the Russian speaking users. As shown in the main paper in the inset of Figure 5, the cumulative size distribution of online support groups for December 30th, 2014, reveals that the system follows a power law (PL) distribution with exponent of -2.5 to 2 significant figures and a p-value of 0.54. For clarity we only show 32 gels in Fig. 2. We follow the same process for the Boogaloos. Figure 1 of the main paper just shows 25 for illustration. The histograms etc. contain the results from all the gels, as do the tests of the power law and determination of the exponent value.

3.1 Statistical analysis of the distribution and power-law test

Our statistical analysis of the distribution follows the strict, state-of-the-art statistical power-law testing procedure laid out by Clauset and co-workers based on maximum likelihood [15, 16, 17]. As proved in Sec. 2 of this SI, our theoretical model predicts a PL size distribution at the transition point with the same exponent $-5/2 = -2.5$, i.e. it is identical to the empirical value to 2 significant figures. We showed this above, i.e. for $t = t_{onset}$ it yields a PL distribution with exponent $-5/2 = -2.5$ that servers as a signal of the dynamical phase transition. On the other hand, for $t < t_{onset}$, the size distribution decays with the size k. Note that for $t > t_{onset}$, the equation is no longer valid since the system has entered into a gel phase and the decomposition discussed earlier should be implemented.

3.2 Gel (or equivalently GCC) onset and growth in size

Since the total number of users and consequently of follows can vary over time, for the individual modeling we take the initial 15-20 days after the movement or cluster (i.e. gel) emerges from a zero population. It is noteworthy that the timescales of the model and the

real data are different and a transformation is required. A timestep in the model is the period where a potential coalescence event occur. We estimate this period in the data by calculating the mean time where a new user follows the movement or cluster (i.e. gel). For example, for ISIS gel 15 the number of follows on a given day (e.g. day 15) after it becomes visible is 865 follows, which over a period of 20 days results in a following rate of $\rho = 43.25$ follows/day, meaning that ρ timesteps in the model are equivalent to one day. The additional days prior the first day in which the gel shows a non-zero population, accounts for the time the gel had remained invisible but showing activity such as sharing a post. Individual gels have the option to turn themselves invisible (e.g. hidden information about actual followers) but they can show activity signal such as posts. The dates of first post detection will vary from gel to gel, hence we use for simplicity a standard of 5 days prior to the first visible date which lies within the average time between the first post and the first visible date. Using this transformation, we are able to estimate that the mean-field aggregation probability associated with gel 15, is given by $F = 0.75$ which implies a mechanism close to that favoring alike individuals; a total population of $N = 1251$; and a gel transition time of:

$$t_c = \frac{N}{2F\rho} = 19.28 \text{ days.}$$

Table 1 contains the statistical fitting parameters calculated for the individual ISIS and Boogaloo gels shown for illustration in Figs. 1 and 2 in the main manuscript.

We now provide evidence to support our claim that the mathematical order that we uncover in the main paper for Boogaloos and ISIS is unlike the results for other collective behaviors of humans online, e.g. fans of a sport, or beverage or the narratives of mature movements such as KKK. We are extremely grateful to Minzhang Zheng for help obtaining these numbers for these other systems. The clump sizes plotted in Fig. S5 over time show specifically that the clumps (i.e. VKontakte Groups) of fans of the mature KKK movement, are remarkably static in terms of membership – unlike the Boogaloos or ISIS in Figs. 1 and 2 of the main paper. Also, if we look at the distribution of sizes for these or other examples, none have the exponent of magnitude 2.5 and most are not even power-law, both of which findings suggest that they show no such dynamical phase transition behavior and hence our results for Boogaloos and ISIS are unexpected and not some everyday typical behavior for humans online. For example, the distribution of clump (i.e. community, or cluster) sizes for the KKK fans is near 1.7; for the anti-vaccine movement on Facebook Pages it is 1.6 and for the pro-vaccination movement it is 1.4. Meanwhile for beer fans in VKontakte Groups, the distribution can be rejected as power-law, as can those discussing Moscow. Meanwhile, VKontakte Groups supporting feminism have an exponent 1.8, for football it is 1.6, for hockey it is 2.0, for New York it is 2.1. It is 2.21 for Facebook Pages of beer fans; 2.3 for New York fans and 1.6 for St. Petersburg fans and the power-law can be rejected. None of these is close to the predicted 2.5 power-law result of our gelation theory. Hence by showing a power-law at the onset of the gel (GCC) transition with exponent near 2.5 – in addition to their highly active size changes over time – our analysis across online activities illustrates that the behaviors of these two extremist movements (Boogaloos and ISIS) is indeed anomalous compared with the clustering associated with other types of human interests.

ISIS group	F	N	SE F	SE N	A r^2
1	1.040	542.600	0.038	4.902	1.000
2	1.020	1152.760	0.040	12.471	1.000
3	1.080	964.670	0.034	2.500	1.000
4	0.990	258.560	0.032	1.380	0.999
5	0.969	1568.680	0.023	18.553	1.000
6	0.833	120.400	0.076	7.700	0.996
7	0.996	570.952	0.024	87.131	0.997
8	0.848	919.028	0.068	75.880	0.996
9	0.988	305.782	0.051	6.069	0.999
10	0.616	543.702	0.055	89.178	0.992
11	0.802	589.600	0.039	11.260	0.998
12	0.862	427.570	0.029	14.315	0.999
13	0.998	653.762	0.034	17.226	0.999
14	0.668	1542.290	0.024	82.131	0.999
15	0.751	1251.080	0.045	92.152	0.997
16	0.577	630.177	0.033	46.800	0.998
17	1.039	309.900	0.037	4.105	0.998
18	0.738	341.288	0.071	35.779	0.993
19	0.862	79.229	0.048	1.750	0.996
20	0.998	113.878	0.064	4.708	0.998
21	0.573	110.135	0.067	16.294	0.986
22	0.477	786.217	0.016	67.121	0.999
23	0.893	466.895	0.043	12.660	0.999
24	0.840	318.481	0.050	18.463	0.997
25	1.083	1518.030	0.060	29.710	0.999
26	0.698	241.654	0.073	32.690	0.990
27	0.950	750.040	0.031	20.136	0.999
28	1.082	63.890	0.150	6.631	0.987
29	0.784	601.536	0.070	57.704	0.995
30	0.719	1338.730	0.041	82.404	0.998
31	0.311	463.514	0.041	119.251	0.993
32	0.980	749.200	0.055	14.800	0.988
Boogaloos					
1	0.342	14868.288	0.002	156.776	0.998
2	0.377	11585.220	0.009	326.412	0.987
3	0.379	2645.597	0.007	56.552	0.995
4	0.435	11807.449	0.004	146.842	1.000
5	0.467	5383.754	0.005	62.906	0.998
6	0.381	15511.488	0.007	342.317	0.991
7	0.392	17876.383	0.005	292.425	0.998
8	0.351	7186.273	0.001	34.146	1.000
9	0.456	5083.861	0.006	82.774	0.999
10	0.354	669.203	0.002	8.464	0.998
11	0.565	5790.489	0.005	61.552	0.999
12	0.445	55272.703	0.004	573.605	1.000
13	0.526	43395.597	0.022	2074.847	0.974
14	0.362	2038.855	0.005	36.225	0.993
15	0.353	11977.511	0.004	212.536	0.996
16	0.351	29799.383	0.007	827.586	0.990
17	0.346	43218.061	0.000	72.414	1.000
18	0.363	41476.372	0.006	909.152	0.991
19	0.685	47438.372	0.010	757.946	0.996
20	0.432	2957.089	0.004	35.868	0.999
21	0.355	7122.731	0.007	188.395	0.987
22	0.357	7628.021	0.003	79.660	0.998
23	0.355	53278.180	0.003	875.147	1.000
24	0.365	10577.164	0.004	139.542	0.993
25	0.457	19310.679	0.002	110.316	1.000

Figure S5: Statistical estimates for individual online gel (i.e. cluster) fitting parameters for ISIS (top) and Boogaloos (below). We use standard least-squares fit. The parameters are: mean-field aggregation probability F, steady-state gel size N, standard error (SE) in F, standard error in N and adjusted R-squared.

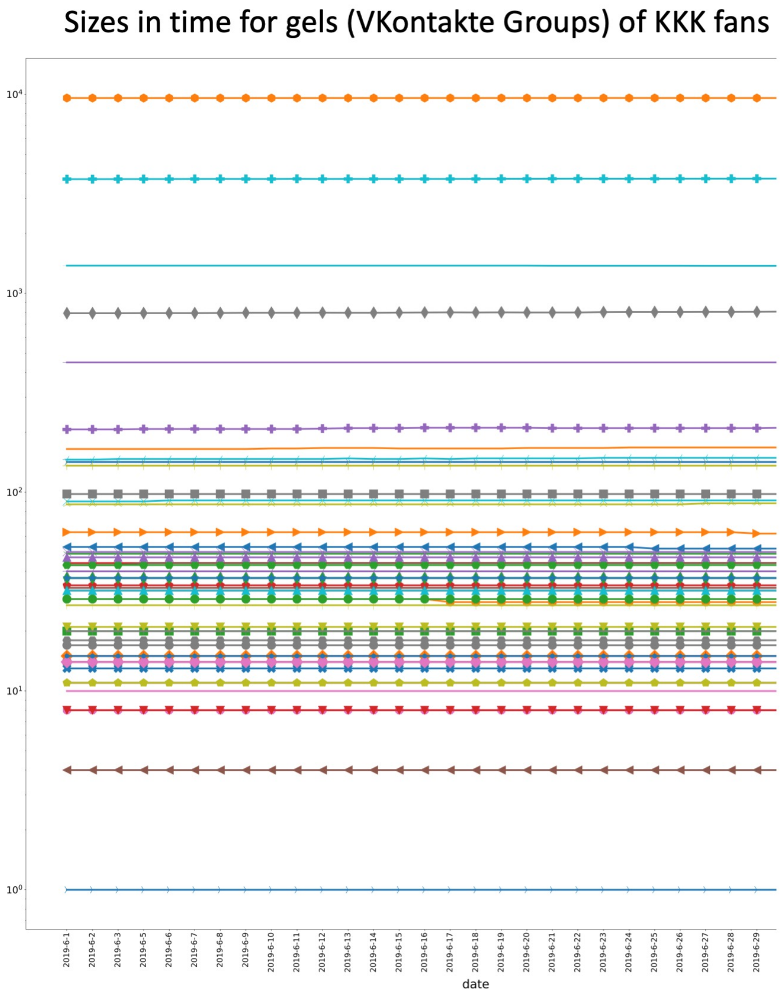

Figure S6: The sizes of KKK communities (i.e. KKK VKontakte Groups) show almost no change over time.

4. Details of Seceder Model in Fig. 6

We only review briefly the analysis and theory underlying the Seceder Model in Fig. 6 since the details are well described in the excellent papers of Halpin-Healy and collaborators, and the original paper of Dittrich et al. in Refs. [18, 19, 20]. Specifically, as explained in detail by Soulier and Halpin-Healy in the case that the character is effectively not along a single dimension, as we sketched in Fig. 6, there are 3 competing populations as in Fig. 6 which are now given by:

$$\frac{\partial L}{\partial t} = L^3 + 3L(R^2 + E^2) + \alpha LRE - L \tag{24}$$

$$\frac{\partial R}{\partial t} = R^3 + 3R(L^2 + E^2) + \beta LRE - R \tag{25}$$

$$\frac{\partial E}{\partial t} = E^3 + 3E(L^2 + R^2) + \gamma LRE - E \tag{26}$$

which are the coupled equations – more precisely, the symmetry-broken replicator equations – for the dynamical evolution of the three branches of the Seceder Model in Ref. [18] where $L, R, E \leq 1$ represent the relative populations of Left, Right and Elsewhere (Boogaloos) and probability conservation imposes the constraint $\alpha + \beta + \gamma = 6$. We refer to Ref. [18] for full details and we summarize their discussion here. In the perfect two-population limit prior to Boogaloos, $\alpha = \beta$ and this yields the fixed points of the dynamics given by $(L, R, E) = (1/2, 1/2, 0)$ and $(L, R, E) = (2/(8-\alpha), 2/(8-\alpha), (4-\alpha)/(8-\alpha))$ which means that for $\alpha < 4$, the 3-population solution with finite Boogaloo movement is stable, and the situation without Boogaloos (i.e. just populations L and R) is unstable. Having $\alpha = \beta = \gamma = 2$ yields $(L, R, E) = (1/3, 1/3, 1/3)$. The simulation in Fig. 6 illustrates the co-existence of these 3 populations, a situation which can arise for a one-dimensional x version in which case $\alpha = \beta = 3$ and $\gamma = 0$ which gives $(L, R, E) = (2/5, 2/5, 1/5)$; and it can also arise for the symmetrical case $\alpha = \beta = \gamma = 2$ which gives $(L, R, E) = (1/3, 1/3, 1/3)$. Figure 3 shows these 3 branches and is characteristic of any situation between, and including, these two extremes.

5. Boogaloos' Topic Coherence in Time

Here we use machine learning analysis of the text in the Boogaloos groups online, to explore how loose the movement is. We find that its coherence score does not increase in time, which is consistent with the idea that they are neither fixed firmly to the far-right or far-left but are 'elsewhere'. The coherence score is described in `https://doi.org/10.1145/2684822.2685324`. A paper that uses the coherence algorithm and explains it well, is `https://doi.org/10.1109/DSAA.2017.61`. The overall coherence score is just a simple

arithmetic mean of all the per-topic coherences. C_v, the coherence score, is based on a sliding window, one-set segmentation of the top words and an indirect confirmation measure that uses normalized point-wise mutual information (NPMI) and the cosine similarity. Essentially, it comprises collections of probability measures on how often top words in topics co-occur with each other in examples of the topics. A link to R. Sear's Github code is `https://github.com/gwclusterlab/ogm`.

In Fig. S7, we show explicitly how the topic coherence of the Boogaloos' community content tends to either decrease in time or stay roughly constant. It does not show any systematic increase. To calculate this, our approach builds on LDA, using dynamical LDA which identifies topics as they evolve in time. The original Blei paper on dynamic topic models is `https://doi.org/10.1145/1143844.1143859`. A reference for using the gensim library is `http://is.muni.cz/publication/884893/en`. Our process is as follows. Step 1: To preprocess the data, we removed special characters from all text, removed stopwords using the stopword list from the gensim package, lemmatized words using the WordNetLemmatizer, then finally stemmed words. Lemmatization and stemming was handled by the nltk package. Any words not recognized by nltk are left unchanged. Lemmatizing is the process of converting all words into a standard form (e.g. "ran," "runs," and "running" are lemmatized into "run"). Stemming is the process of removing endings from words (e.g. "apples" is stemmed to "apple"). Step 2: We applied Gensim's Sequential LDA model (an implementation of a dynamic topic model) to the preprocessed posts. We trained Sequential LDA models with the the n_topics parameter ranging from 3-10. Step 3: We evaluated the goodness of fit for each LDA model using the CV coherence score algorithm.

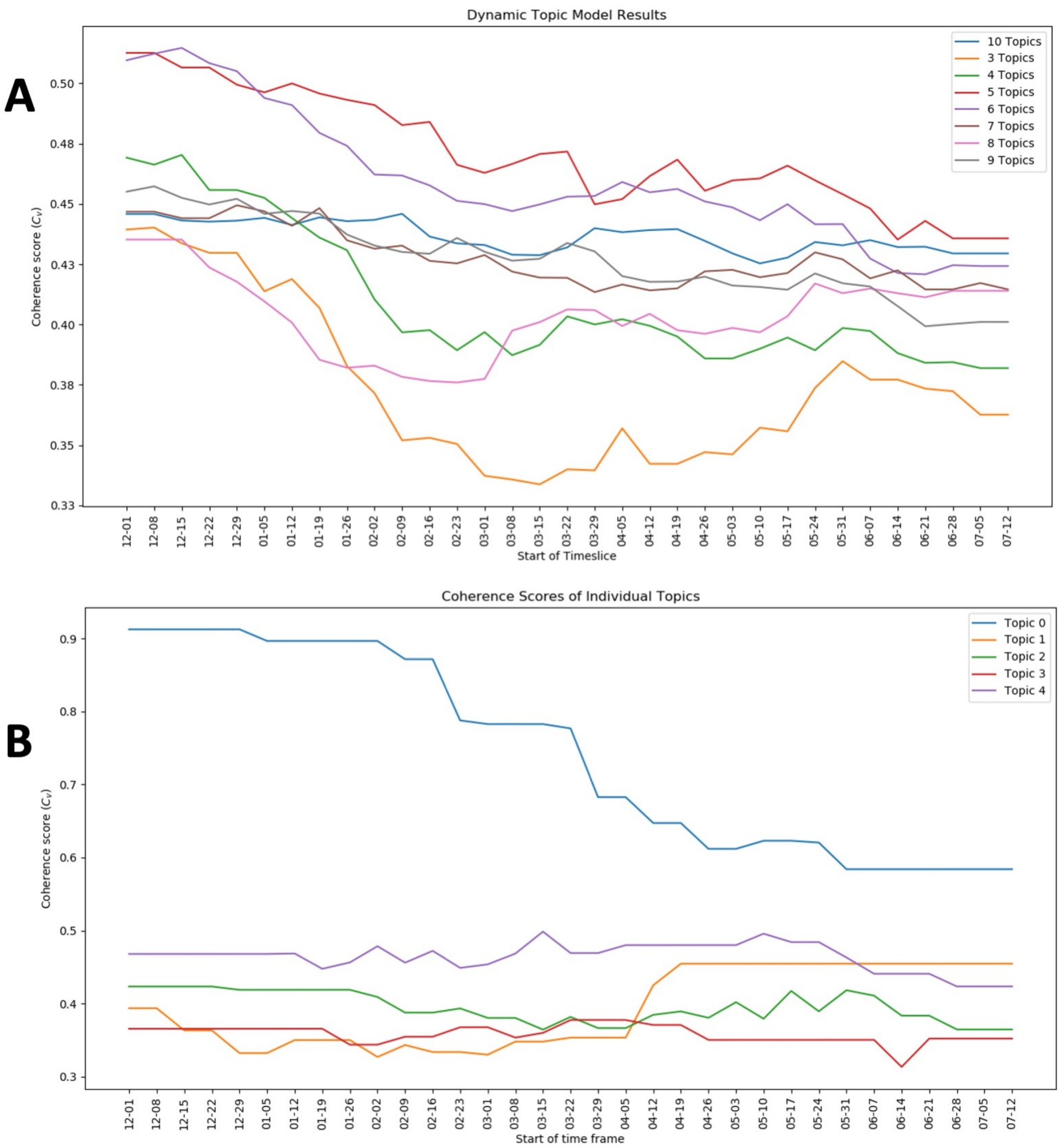

Figure S7: Topic coherence of the Boogaloos' community content tends to either decrease in time or stay roughly constant. It does not show any systematic increase. A shows the average coherence score for a particular number of topics, versus time. B shows the score per topic for the number of topics that tends to have the highest coherence score from panel A. Details of the coherence score and dynamic LDA are given in the text.

References

[1] P. L. Krapivsky, S. Redner and E. Ben-Naim *A Kinetic View of Statistical Physics*, (Cambridge University Press, Cambridge, 2010).

[2] N. F. Johnson, M. Zheng, Y. Vorobyeva, A. Gabriel, N. Velasquez, P. Manrique, D. Johnson, E. Restrepo, C. Song and S. Wuchty. *Science* **352**, 6292, 1459-1463 (2016)

[3] G. Palla, A., Barabási, T. Vicsek. Quantifying social group evolution. *Nature* **446**, 664–667 (2007)

[4] R. L. Drake, *Topics in Current Aerosol Research*, Vol. 3, G. M. Hidy and J. R. Brock, eds. (Pergamom Press, New York, 1972), Part 2.

[5] M. H. Ernst, *Fractals in Physics*, L. Pietronero and E. Tosatti, eds. (North-Holland, Amsterdam, 1986), p.289

[6] N.F. Johnson, P. Manrique and P. M. Hui. *J. Stat. Phys.* **151**, 395 (2013)

[7] P.D. Manrique, P. M. Hui and N. F. Johnson. *Phys. Rev. E* **92**, 062803 (2015)

[8] P. G. J. van Dongen and M. H. Ernst. *J. Stat. Phys.* **49**, 889-926 (1987)

[9] P.J. Flory. Molecular Size Distribution in Three Dimensional Polymers. I. Gelation. *J. Am. Chem. Soc.* **63**, 3083, (1941)

[10] W.H. Stockmayer. Theory of Molecular Size Distribution and Gel Formation in Branched Polymers II. General Cross Linking. *Journal of Chemical Physics.* **12**,4, 125, (1944)

[11] D. Stauffer, & Aharony, A. *Introduction to Percolation Theory* (Taylor & Francis, 1994)

[12] M. Sahimi, *Applications of Percolation Theory* (Taylor & Francis, 1994)

[13] P. Erdös, & Rényi, A. On random graphs I. Math. *Debrecen* **6**, 290-297 (1959)

[14] P. Erdös, & Rényi, A. On the evolution of random graphs *Publ. Math. Inst. Hungar. Acad. Sci.* **5**, 17-61 (1960).

[15] J.C. Bohorquez, et al. Common ecology quantifies human insurgency. Nature **462**, 911–914 (2009).

[16] A. Clauset, Young, M. & Gleditsch, K. S. On the frequency of severe terrorist events. J. Confl. Resolut. **51**, 58–87 (2007).

[17] A. Clauset, & Gleditsch, K. The Developmental Dynamics of Terrorist Organizations. *PLoS One* **7**, e48633 (2012).

[18] A. Soulier, T. Halpin-Healy. Population fragmentation and party dynamics in an evolutionary political game. arXiv:cond-mat/0305356v1 [cond-mat.stat-mech] 15 May 2003

[19] A. Soulier, T. Halpin-Healy. The Dynamics of Multidimensional Secession: Fixed Points and Ideological Condensation. Phys. Rev. Lett. **90**, 258103 (2003)

[20] P. Dittrich, F. Liljeros, A. Soulier, W. Banzhaf. Spontaneous Group Formation in the Seceder Model. Phys. Rev. Lett. **84**, 3205 (2000)
